# Geo-spatial prospective life cycle sustainability of InGaN and InGaP compound semiconductors

**DOI:** 10.1038/s41598-026-43622-5

**Published:** 2026-03-16

**Authors:** Moein Shamoushaki, Josie Travers-Nabialek, Sara-Jayne Gillgrass, Peter M. Smowton, S. C. Lenny Koh

**Affiliations:** 1 Sheffield University Management School, The University of Sheffield, Sheffield, S10 1FL UK; 2 Energy Institute, The University of Sheffield, Sheffield, S10 2TN UK; 3 School of Physics and Astronomy, Cardiff University, Cardiff, CF24 3AA UK

**Keywords:** Chemistry, Environmental sciences, Materials science

## Abstract

This is the first study which presents integrated geo-spatial prospective life cycle and supply chain sustainability modelling of two compound semiconductors: Indium Gallium Nitride (InGaN) and Indium Gallium Phosphide (InGaP), in 80 international supply chain scenarios, incorporating 11 countries across 4-time horizons—024, 2030, 2040 and 2050. The results show environmental sustainability is geographic- and time- dependent and varied by properties of the supply chain characteristics. The manufacturing of InGaN and InGaP excel in UK based scenarios (~ 70% and 66% impact reduction from 2040 to 2050). Scenarios involving shared fabrication in the UK and US show strong sustainability performance in 2024, those for fabrication in Taiwan (for both materials) and US (InGaN) demonstrate increasing sustainability potential by 2050. Scenarios involving fabrication in China consistently led to a higher environmental impact. However, all 80 configurations demonstrate marked reductions in environmental impacts, primarily due to global electricity grid decarbonisation, and improved emissions controls. Despite this improvement in the clean room energy impact, epitaxial growth and substrate preparation remain the hotspots, calling for process innovation, cleaner precursors (e.g., replacing arsine or phosphine), and advanced material recycling. InGaN generally performs better than InGaP in most categories, attributed to its simpler material inputs and lower toxicity potential. InGaP scenarios exhibit higher marine ecotoxicity, carcinogenic toxicity, and mineral resource scarcity, driven by complex chemistries and Gallium Arsenide (GaAs) substrates. Interestingly, InGaP scenarios significantly outperform InGaN in stratospheric ozone depletion due to limited use of halogenated chemicals. This study provides compelling evidence to support reshoring or nearshoring of compound semiconductor fabrication to regions with cleaner energy profiles and stronger environmental regulations. Scenarios involving the UK, USA and Taiwan (specially in 2050), consistently achieve higher sustainability scores across global warming, toxicity, and resource depletion categories.

**Supplementary Information:**

The online version contains supplementary material available at 10.1038/s41598-026-43622-5.

## Introduction

The supply chain (SC) framework is envisioned as a value chain network comprising separate functional units that collaborate to deliver resources and share information. This integration aims to optimise supplier management and ensure the efficient flow of components throughout the system^1^. Over the past decades, green and sustainable SC management practices have been developed to integrate environmental concerns into organisational operations, with the goal of reducing the unintended negative environmental effects of production and consumption activities^2^. This paradigm shift towards sustainability has sparked discussions on how to balance environmental objectives with the need to strengthen the structural resilience of SCs, particularly in critical industries like compound semiconductors. Today’s central discussion in the global semiconductor SC focuses on its structural resilience and potential vulnerabilities. The semiconductor industry’s SC constitutes a highly intricate network involving numerous stakeholders and processes, spanning the design, manufacturing, testing, and distribution of semiconductor products^3^.

As semiconductor technology rapidly advances, its applications have broadened from smartphones to cloud computing and have extended to artificial intelligence systems, all of which depend on semiconductor components^4^. The semiconductor industry, central to modern technology and global progress, underpins sectors like consumer electronics and automotive. However, rising demand and SC disruptions, from raw materials to product delivery, now pose serious challenges to its stability^5^. However, the semiconductor industry’s consumption of energy and other resources is steadily rising due to the increasing complexity of wafer processing and the global expansion of production. Despite the promise of new technologies as a weapon against climate change, the present semiconductor production method is not environmentally friendly^6^. Therefore, in building new semiconductor manufacturing capacity, it is crucial to prioritise reducing emissions and improving sustainability^7^. The industries advance environmental sustainability by cutting energy use, reducing perfluorocarbons (PFC) emissions, and boosting energy efficiency. Managing low-carbon SCs has emerged as a key strategy for promoting sustainable development in the semiconductor industry^8^.

Given the growing significance of environmental concerns, numerous companies and research institutions (e.g., IQE) are enhancing their sustainable development practices. This shift is driven by the influence of environmental regulatory agencies and amplified by mass media, which underscores the global commitment to mitigating environmental impacts. The ultimate aim is to preserve ecosystem quality and promote an improved quality of life for all living beings^9^. A highly effective and practical approach involves employing Life Cycle Assessment (LCA) to identify key hotspots and critical processes, enabling the simultaneous reduction of environmental impacts and advancement of technological development. However, conducting life cycle SC assessment of compound semiconductor industry is particularly challenging. To the best of the authors’ knowledge, no comprehensive LCA data currently exists specifically addressing Wide Band Gap (WBG) semiconductors. This scarcity is attributable to the complexity of manufacturing processes, which involve numerous steps, the use of specialty chemicals, energy-intensive cleanroom environments, and rapid technological advancements within a highly competitive and protective industry. Additionally, most existing semiconductor SC research primarily focuses on silicon-based technologies rather than WBG semiconductors^10^. The bandgap is a fundamental property that defines the electrical and optical characteristics of semiconductors^11^. Wide bandgap semiconductors exhibit exceptional material properties, offering the potential to deliver performance improvements by orders of magnitude beyond current achievements^12^. Wide bandgap semiconductors, characterized by bandgap energies at least three times larger than silicon, can withstand substantially higher voltages. This allows for smaller device sizes, enabling faster switching with lower resistance. The reduced resistance minimizes energy loss as heat, enhancing the energy efficiency of wide bandgap devices compared to their silicon counterparts^10^. III–V semiconductors, especially those incorporating indium and gallium, have been widely regarded as auspicious materials for diverse electronic and optoelectronic applications in the future^13^. The flexibility of III–V semiconductors often means that there are different options for satisfying particular application requirements. With a functioning LCA, choices on approach can be based on the environmental and SC implications.

A major challenge facing the commercialisation of red-green-blue (RGB) micro-LED (Light-Emitting Diode) displays is the ability to develop a fully-integrated platform containing all three colours with the same micron-sized footprint and which operate with comparable brightness, efficiency and longevity^14^. Two feasible approaches to realising this technology include the monolithic integration of RGB pixels processed from a single InGaN/GaN (Indium Gallium Nitride/Gallium Nitride) epitaxial wafer^15^ or, alternatively, using transfer printing techniques to heterogeneously integrate InGaN green/blue LEDs with AlGaInP/InGaP (Aluminium Gallium Indium Phosphide/Indium Gallium Phosphide) (hereafter referred to as InGaP throughout the manuscript)-based red-emitters^16^.

Despite III-nitride green/blue LEDs successfully achieving external quantum efficiencies (EQE) of up to 56% and 84% ^17,18^, respectively, the EQE of InGaN red devices is considerably less (< 10%) ^19^. To extend the emission wavelength into the red region (620–630 nm) more than 30% indium (In) must be incorporated into the InGaN quantum wells (QWs). To grow high In-content QWs, relatively low-temperature epitaxy is required to overcome the large lattice mismatch between GaN and InN but such growth conditions typically produce a crystal lattice with a high defect density which subsequently leads to wavelength instability and a rapid decline in the efficiency and lifetime of processed LEDs. Additionally, the quantum-confined Stark effect (QCSE) becomes significant in high In-content layers which lowers the spontaneous recombination rate within the QWs and reduces the LED light output as a consequence. Unlike red InGaN LEDs, native red-emitting InGaP devices are known for their low defect densities and large area mesa EQEs of up to 50%^20^, owing to the well-established epitaxial growth on gallium arsenide (GaAs) substrates. However, the EQE of the LED decreases rapidly as the structure shrinks to mesa sizes of < 10 μm due to long carrier diffusion lengths associated with phosphide-based alloys which contribute to an increase in nonradiative losses at the etched sidewalls^21^. To overcome size-dependent efficiency droop in the InGaP LEDs, additional processing steps such as dielectric passivation and chemical solution treatments have been developed to suppress sidewall defects^22,23^.

Whilst research efforts continue to realise the smallest and brightest pixels-per-square-inch for the next generation of high-resolution display applications, the efficiency and lifetime of current systems still pose challenges and what remains unclear is which aforementioned approach to this technology is the most sustainable; from both an environmental and economic perspective. Processing RGB LEDs on a single GaN wafer benefits the environment by reducing material usage/waste and removes the need for arsenic-containing compounds typically used in the heterogeneous approach, however the EQE of the red InGaN LED is still not comparable to the InGaP equivalent and therefore could waste considerable energy during the LED’s operational phase. Although, InGaP red LEDs are able to deliver acceptable EQE values this is not without additional processing steps that will inevitably consume further energy, water and materials. A separate growth process is required to produce the InGaP micro-LEDs due to the incompatibility between the InGaP and InGaN material systems which could produce further waste unless the substrate can be recycled/reclaimed. Some studies examined the physics and technical aspects of semiconductors to boost efficiency, improve performance and optimise material use, directly and indirectly reducing the ecological footprint^24–33^.

Growing research has applied LCA approaches to examine the environmental implications of semiconductor manufacturing processes, focusing on material extraction, energy consumption, and emissions across the product life cycle. Development of parametric carbon footprinting tools for the semiconductor industry facilitates more accurate and scalable assessment of process-related emissions^34^. Critical review of LED lamp LCAs reveals significant data gaps and inconsistencies that hinder accurate assessment of environmental impacts in electronics manufacturing^35^. The urgent need to address the growing electronic waste problem through circular economy strategies was highlighted, with emphasis placed on repair, reuse, and responsible recycling^36^. Ex ante LCA of GaAs/Si nanowire tandem solar cells indicates that early design choices in semiconductor processing critically influence future environmental performance and scalability^37^. Variability in LCA results for electronics is largely driven by differences in data quality, system boundaries, and assumptions about semiconductor manufacturing processes^38^. Decarbonising semiconductor manufacturing requires a multifaceted approach, including electrification, renewable energy integration, and mitigation of high-GWP gases used in fabrication^39^. Cradle-to-gate LCA of GaN power devices reveals that process gases and energy-intensive steps, particularly epitaxy and wafer processing, dominate the environmental impacts^10^. Despite efficiency gains, PFC emissions remain a major environmental concern in the semiconductor foundry, emphasising the need for cleaner processes and effective abatement technologies^40^.

Some studies have examined semiconductor production within the context of environmental impacts and SCs to identify ways to reduce resource use and emissions. Upstream SCs in electronics manufacturing significantly contribute to the overall carbon footprint, highlighting the need for SC-focused emission reduction strategies^41^. Carbon pricing significantly influences the semiconductor industry’s green SC integration by incentivising emission reductions and promoting sustainable practices^42^. Resource compensation and complete information sharing are key drivers for enhancing green sustainability in semiconductor SCs^43^. Sustainable SC transparency in the semiconductor industry is critical for improving environmental performance and stakeholder trust through hierarchical interdependence management^3^. Limited studies examine the environmental impacts of compound semiconductor (especially WBG) from SC perspective. No research models and predicts the compound semiconductor SC sustainability inter-dependency of geography and time over a geo-spatial perspective representing leaders of the semiconductor industry.

This study breaks new ground by addressing these key gaps—specifically for InGaN and InGaP - used in LED technologies.

It aims to answer 2 key research questions: (1) how do properties of compound semiconductor (in this case InGaN and InGaP) SCs characterise their sustainability; (2) why sustainable sourcing and manufacturing of compound semiconductor (in this case InGaN and InGaP) are geographic- and time-dependent? What sets this study apart is its novel integration of geo-spatial prospective LCA framework, for the first time at a global scale to 80 compound semiconductor SCs involving 11 countries. By combining spatial data on international supply networks with forward-looking LCA projections, our research predicts 18 types of environmental impacts across 3 critical future milestones − 2030, 2040, and 2050 - reflecting evolving energy systems shaped by increasing shares of renewable electricity generation aligned with global net-zero targets, against the baseline of 2024. This approach provides a dynamic and granular understanding of how properties shift in the energy mix and SC configurations influence the ecological footprint of these advanced materials. By modelling key upstream raw material suppliers, major fabrication sites, and downstream testing and packaging processes in our geo-spatial prospective LCA models, the study identifies and ranks significant environmental hotspots and sustainable SCs, offers actionable insights to industry players and policymakers aiming to integrate sustainability while fostering innovation.

This geospatially informed method provides a unique and location-sensitive perspective that has not been explored previously. This is the first comprehensive, geo-spatially informed, prospective LCA of InGaN and GaInP technologies for LED applications - pioneering a holistic, future-oriented pathway to support the sustainable development of compound semiconductors that are vital for next-generation electronic and photonic devices.

### Semiconductor supply chain and developed scenarios

The compound semiconductor industry and SC heavily rely on critical materials such as gallium (Ga), indium (In), and arsenic (As), where availability and resilience are vulnerable to disruptions such as geopolitical tensions, regulatory shifts, and environmental degradation. Additionally, the production of these semiconductors depends on essential mineral resources like Ga and germanium (Ge). Given the geographical concentration of these resources, countries with limited natural reserves are compelled to depend significantly on trade imports to maintain their semiconductor industries^4^. The semiconductor SC is a multifaceted network of organisations and processes involved in producing and delivering semiconductor components, covering design, manufacturing, assembly, packaging, testing, and distribution^44^.

**Fig. 1:**
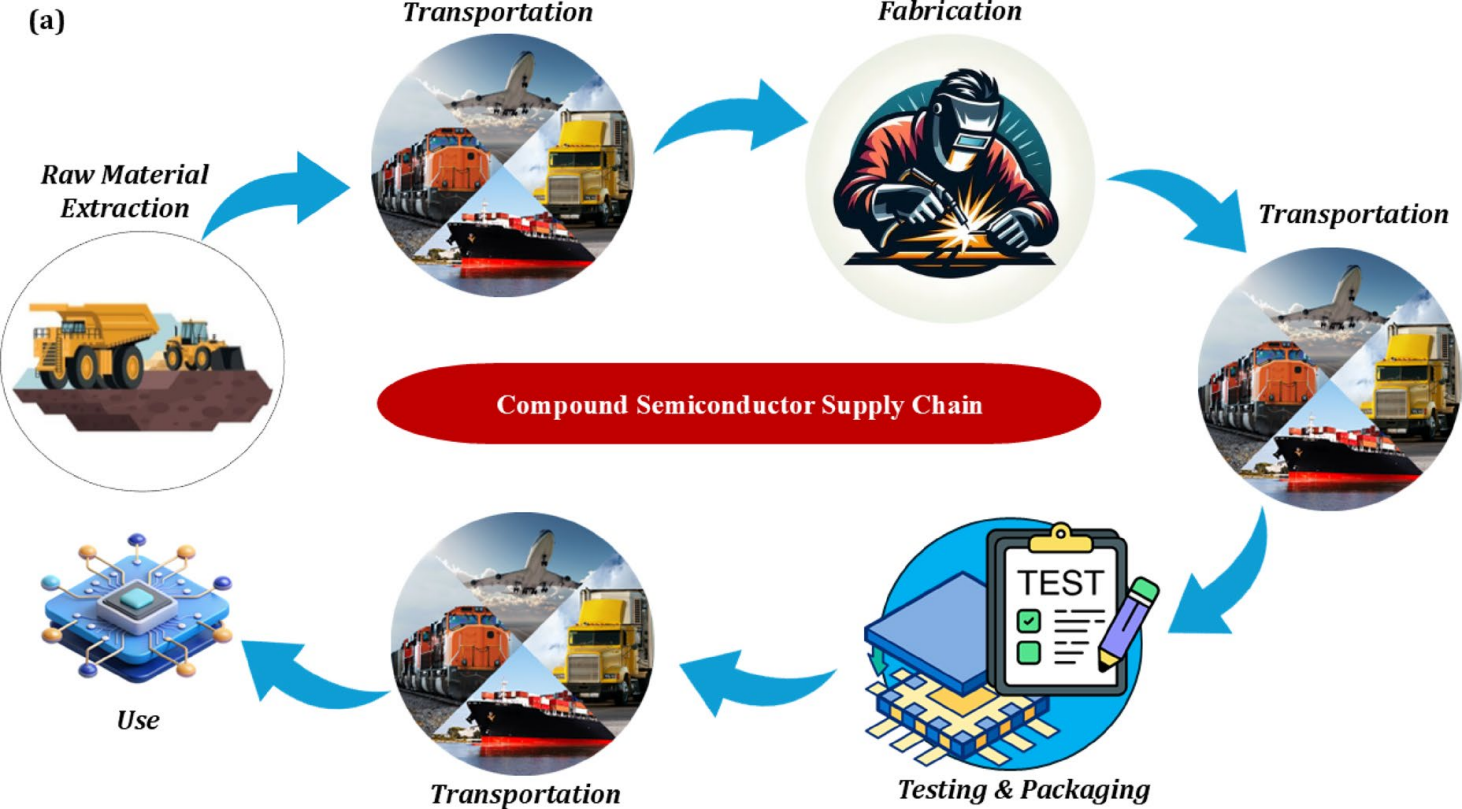

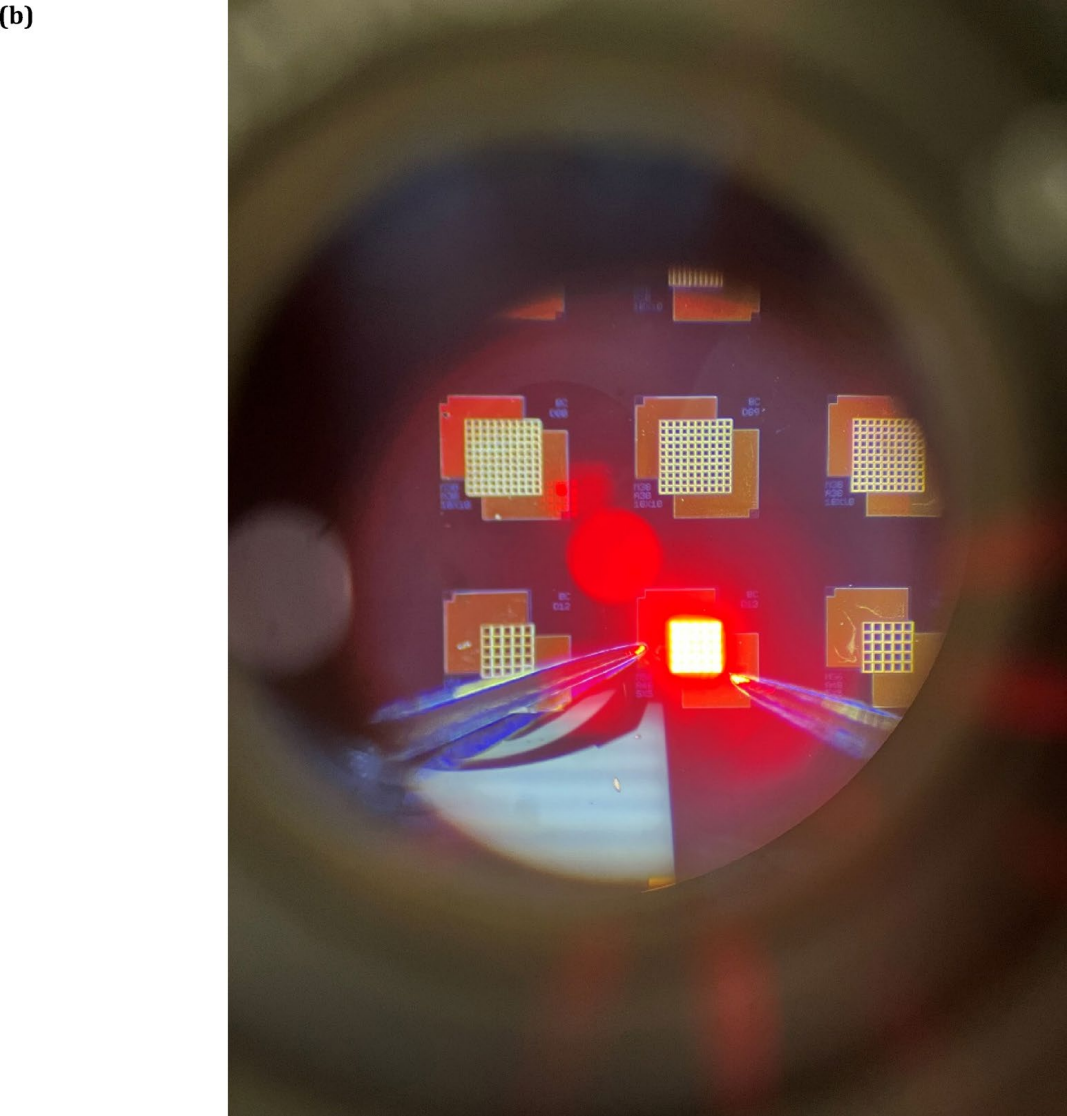
(**a**) Compound semiconductor supply chain, (**b**) Microscope view of a fabricated GaInP LED array under electrical operation.

To assess the environmental performance and strategic feasibility of compound semiconductor manufacturing, we developed a total of 80 distinct international SC scenarios (40 for each technology) for InGaN and InGaP. These scenarios are distributed across four key timeframes − 2024, 2030, 2040, and 2050 - reflecting the evolving net-zero targets and decarbonisation trajectories over the next 30 years. Each scenario is labelled using a structured format: “N-SX” refers to InGaN scenarios, while “P-SX” designates InGaP scenarios, where “S” stands for ‘Scenario’ and “X” is the scenario number (e.g., N-S1 to N-S10 and P-S1 to P-S10). The SCs are designed across four main phases: raw material extraction and supply, fabrication, testing and packaging, and end-use (Fig. 1). Critical raw materials (Such as Ga, In, and Phosphorus (P, specifically for InGaP)) are carefully tracked across sourcing countries, while less critical materials are assumed to be locally available or sourced with minimal SC effort.

Raw material supply locations were chosen based on current global production, strategic importance, and geopolitical considerations. China, the world largest producer of Ga and In, is featured prominently due to its dominant role in critical mineral supply including Ga and In extraction, while South Korea, Canada, Morocco (for Phosphorus) and Russia are included to model potential alternative sources. Fabrication hubs are distributed among countries with strong semiconductor industries, including Taiwan, South Korea, China, Japan, the USA, and the UK. Testing and packaging locations are selected either to be co-located with fabrication sites or placed in countries known for back-end semiconductor specialisation, notably Malaysia and Taiwan, which are globally recognised for their expertise and infrastructure in this phase. End-use regions, such as the UK, Germany, Japan, China, and the USA, are selected based on their advanced electronics markets and growing demand for compound semiconductors. This scenario-based design allows for comprehensive analysis of sustainability trade-offs, geopolitical risks, and carbon footprints associated with each global configuration over time. The summary of modelled SCs is presented in Table 1.

**Table 1:** Scenario designations and assessment horizons for InGaN and InGaP semiconductor supply chains. Scenarios for InGaN are denoted as N-S1 to N-S10, and scenarios for InGaP as P-S1 to P-S10. Each scenario represents a distinct supply chain configuration and was evaluated across four timeframes: 2024, 2030, 2040, and 2050. In total, 80 scenarios were assessed: 10 scenarios for each material and each timeframe (10 × 4 = 40 for InGaN; 10 × 4 = 40 for InGaP). This naming convention distinguishes material type (N = InGaN, P = InGaP), scenario number (S1–S10), and the year of assessment.

Scenario	Raw material	Transportation	Fabrication	Transportation	Testing & Packaging	Transportation	Use
**InGaN**							
N-S1	China (Ga & In)	Lorry & Sea freight	Taiwan	Air Freight	Taiwan	Air Freight	UK
N-S2	China (Ga & In)	Lorry & Sea freight	China	Air Freight	China	Air Freight	China
N-S3	China (Ga) & South Korea (In)	Lorry & Sea freight	UK	Air Freight	Malaysia	Air Freight	Japan
N-S4	China (Ga) & Canada (In)	Lorry & Sea freight	USA	Air Freight	USA	Air Freight	USA
N-S5	China (Ga) & South Korea (In)	Lorry & Sea freight	South Korea	Air Freight	China	Air Freight	Germany
N-S6	Russia (Ga) & China (In)	Lorry & Sea freight	Taiwan (50%) - South Korea (50%)	Air Freight	Malaysia	Air Freight	China
N-S7	Russia (Ga) & South Korea (In)	Lorry & Sea freight	China (50%) - Japan (50%)	Air Freight	Taiwan	Air Freight	USA
N-S8	China (Ga) & Canada (In)	Lorry & Sea freight	UK (50%) - USA (50%)	Air Freight	UK	Air Freight	Germany
N-S9	China (Ga) & China (In)	Lorry & Sea freight	USA (50%) - China (50%)	Air Freight	China	Air Freight	Taiwan
N-S10	China (Ga) & South Korea (In)	Lorry & Sea freight	Japan (50%) - Taiwan (50%)	Air Freight	Taiwan	Air Freight	UK
**InGaP**							
P-S1	China (Ga & In) & Morroco (P)	Lorry & Sea freight	Taiwan	Air Freight	Taiwan	Air Freight	UK
P-S2	China (Ga & In) & Morroco (P)	Lorry & Sea freight	China	Air Freight	China	Air Freight	China
P-S3	China (Ga) & South Korea (In) & China (P)	Lorry & Sea freight	UK	Air Freight	Malaysia	Air Freight	Japan
P-S4	China (Ga) & Canada (In) & China (P)	Lorry & Sea freight	USA	Air Freight	USA	Air Freight	USA
P-S5	China (Ga) & South Korea (In) & Morroco (P)	Lorry & Sea freight	South Korea	Air Freight	China	Air Freight	Germany
P-S6	Russia (Ga) & China (In) & Morroco (P)	Lorry & Sea freight	Taiwan (50%) - South Korea (50%)	Air Freight	Malaysia	Air Freight	China
P-S7	Russia (Ga) & South Korea (In) & China (P)	Lorry & Sea freight	China (50%) - Japan (50%)	Air Freight	Taiwan	Air Freight	USA
P-S8	China (Ga) & Canada (In) & China (P)	Lorry & Sea freight	UK (50%) - USA (50%)	Air Freight	UK	Air Freight	Germany
P-S9	China (Ga) & China (In) & Morroco (P)	Lorry & Sea freight	USA (50%) - China (50%)	Air Freight	China	Air Freight	Taiwan
P-S10	China (Ga) & South Korea (In) & Morroco (P)	Lorry & Sea freight	Japan (50%) - Taiwan (50%)	Air Freight	Taiwan	Air Freight	UK

## Results

### Geo-spatial prospective LCA

**Fig. 2:**
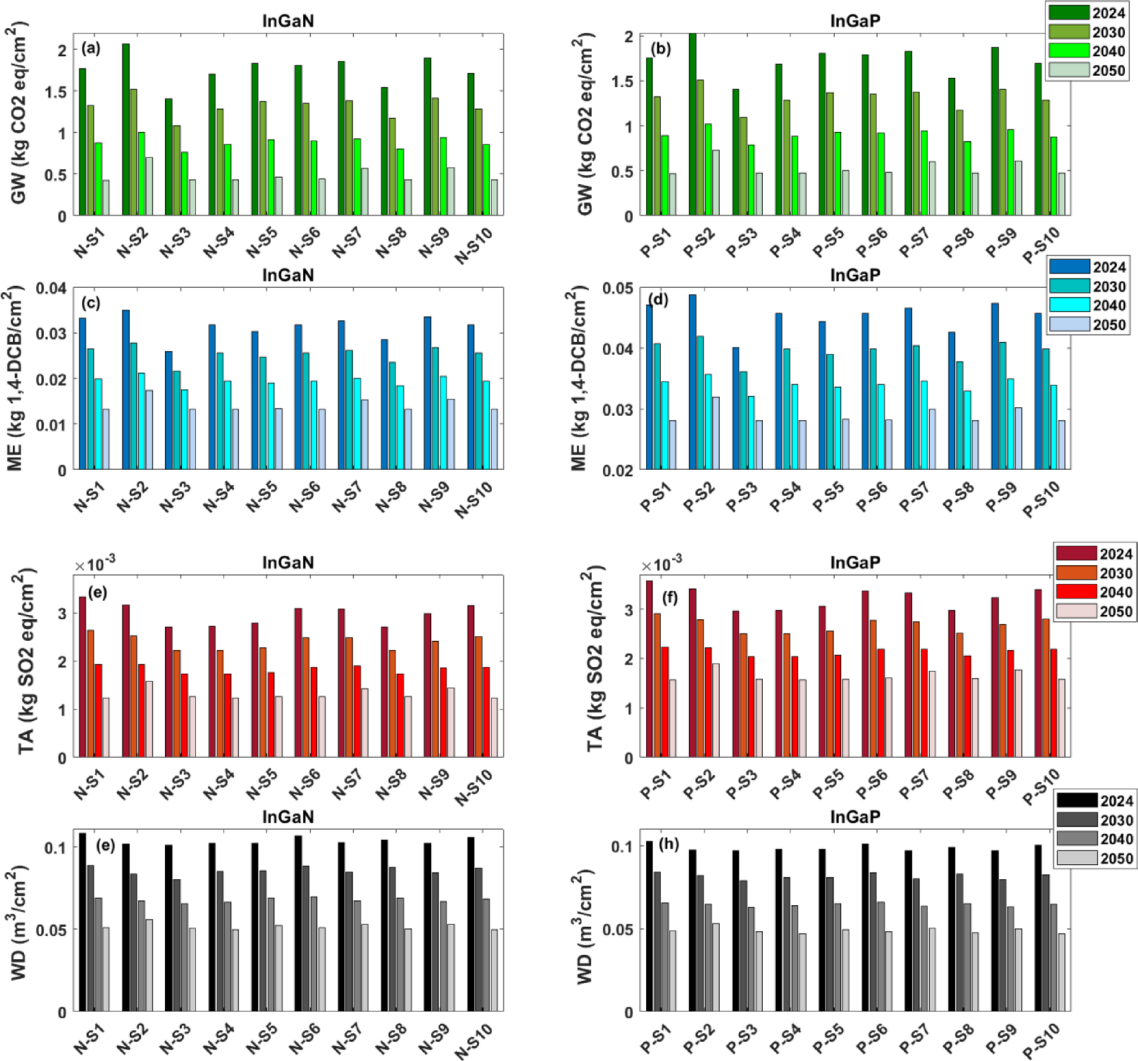
Environmental pollution variations across four impact categories for all studied compound semiconductor supply chain scenarios, in alignment with the Net Zero plan over 2024, 2030, 2040 and 2050, (**a**) GW for InGaN supply chains, (**b**) GW for InGaP supply chains, (**c**) ME for InGaN supply chains, (**d**) ME for InGaP supply chains, (**e**) TA for InGaN supply chains, (**f**) TA for InGaP supply chains, (**g**) WD for InGaN supply chains, and (**h**) WD for InGaP supply chains.

Figure 2 presents geo-spatial prospective LCA results for InGaN and InGaP semiconductors across 80 SC configurations from 2024 to 2050. The model incorporates regional decarbonisation pathways aligned with national net-zero targets, especially in electricity generation, which dominates total emissions along with some material inputs used in fabrication.

#### Global warming (GW)

*Temporal Trends*: Both InGaN and InGaP show steady carbon intensity reductions over time across all scenarios, driven by decreasing fossil fuel use in electricity mixes of countries (Fig. 2a,b). For instance, Scenario N-S1 (fabrication, testing and packaging in Taiwan) shows InGaN’s GW impact falling from 1.77 to 0.42 kg CO₂ eq/cm² between 2024 and 2050. InGaP follows a similar trend.

*Scenario Comparison (2024 Baseline)*: Highest GW impacts in 2024 occur in Scenarios N-S2 and P-S2 (fabrication and testing in coal-dependent China), with InGaN at 2.07 and InGaP at 2.03 kg CO₂ eq/cm². Scenario 3 (UK fabrication, Malaysia testing and packaging) has the lowest impacts due to the UK’s cleaner energy mix, despite some added environmental burden from Malaysian testing.

*Geographic Influence and SC Design*: High-carbon grid regions (China, Taiwan, South Korea) in fabrication yield higher impacts, e.g., Scenario N-S9 and P-S9 (USA-China fabrication, China testing and packaging) results in elevated emissions. Conversely, scenarios with fabrication in lower-carbon regions (UK, USA) consistently perform better, highlighting the importance of clean electricity in energy-intensive steps.

*Material Contribution and Process Sensitivity*: Electricity dominates the GW impact, though fabrication materials, especially precursor gases used in epitaxial growth, also contribute. InGaP generally shows slightly lower GW impacts because its epitaxial growth is carried out at lower temperatures than that of InGaN, which reduces electricity demand.

*Long-term Perspective (2050)*: By 2050, emissions across scenarios converge (~ 0.42–0.58 kg CO₂ eq/cm² for InGaN and ~ 0.46–0.61 for InGaP), reflecting global decarbonisation. However, scenarios involving China remain highest, while Scenarios N-S1, P-S1, N-S3, P-S3, N-S8 and P-S8 achieve lowest emissions, indicating the most favourable outcomes.

#### Marine ecotoxicity (ME)

Figure 2c,d show ME impacts for InGaN and InGaP under scenarios from 2024 to 2050. ME reflects ecotoxicological burdens from heavy metals and toxic substances released into aquatic systems, driven by coal-based electricity, chemical inputs, and regional pollution controls.

*Temporal Trends*: ME impacts decline over time, paralleling electricity grid decarbonisation and stricter regulations. For instance, in Scenario N-S1, InGaN’s ME impact drops from 0.0331 to 0.0133 kg 1,4-DCB eq/cm² by 2050; InGaP decreases similarly but remains higher.

*Scenario Comparison (2024 Baseline)*: Scenario P-S2 (China fabrication, testing and packaging) has the highest ME impacts due to coal-fired emissions. Scenario N-S3 (UK fabrication, Malaysia testing and packaging) shows the lowest ME, benefiting from cleaner grids and sustainable chemical management.

*Geographic Influence and SC Design*: Fabrication in coal-heavy grid countries leads to higher ME impacts. Partial fabrication in the USA (Scenarios N-S9 and P-S9) does not offset the effect of China’s emissions. Scenarios with UK and USA fabrication have consistently lower ME due to cleaner energy and environmental governance.

*Material Contribution and Process Sensitivity*: Unlike GW, ME is strongly affected by substrate type (sapphire for InGaN, GaAs for InGaP) and hazardous precursor chemicals (arsine, phosphine, metalorganics). Regional wastewater treatment quality also influences ME impacts.

*Long-term Perspective (2050)*: ME impacts decline but remain differentiated by substrate and chemical use. InGaP’s ME remains higher than InGaN’s. Scenario N-S2 and P-S2 remain highest, while Scenarios N-S3, P-S3, N-S8and P-S8 maintain the lowest impacts, emphasising the benefits of clean energy and strong regulations.

#### Terrestrial acidification (TA)

Figure 2e,f show TA impacts for InGaN and InGaP for all scenarios, reflecting acidifying emissions like SO₂, NOₓ, and NH₃ mainly from fossil fuel power and chemical synthesis.

*Temporal Trends*: TA impacts decrease over time with grid decarbonisation and improved pollution controls. For example, Scenario N-S1’s InGaN TA drops from 0.00334 to 0.00123 kg SO₂ eq/cm² between 2024 and 2050.

*Scenario Comparison (2024 Baseline)*: Highest TA impacts in 2024 occur in Scenario 1 (Taiwan fabrication, testing and packaging) and Scenario 2 (China fabrication, testing and packaging), linked to coal-heavy grids. The UK fabrication-Malaysia testing and packaging (Scenarios N-S3 and P-S3) and UK-USA fabrication—UK testing and packaging (Scenarios N-S8 and P-S8) scenarios show the lowest TA due to cleaner energy mixes.

*Geographic Influence and SC Design*: TA is sensitive to both energy source and precursor emissions. Coal-dependent regions produce higher acidification, especially in fabrication. Fabrication in countries with stricter emissions controls will lessen the TA impacts.

*Chemical Contribution and Process Sensitivity*: TA is influenced by metal-organic precursors used in Metal-Organic Chemical Vapor Deposition (MOCVD) (e.g., Trimethylgallium (TMGa), Trimethylindium (TMIn), Trimethylaluminum (TMAl)), which can emit acid-forming substances. InGaP shows slightly higher TA than InGaN, likely due to process differences and greater material use.

*Long-term Perspective (2050)*: TA impacts reduce significantly and converge across scenarios. Scenario 2 remains highest, while Scenarios N-S3, P-S3, N-S8 and P-S8 continue to show the lowest values.

#### Water Depletion (WD)

Figure 2g,h illustrate WD impacts (m³ water eq/cm²) for both compound semiconductors’ technologies across all SC scenarios.

*Temporal Trends*: Water depletion impacts decline moderately over time, influenced by improvements in water use efficiency and energy mix changes. Unlike GW and ME, WD is less sensitive to decarbonisation alone due to substantial process water demands.

*Scenario Comparison (2024 Baseline)*: Scenarios N-S1 and P-S1 (Taiwan fabrication, testing and packaging) show the highest WD impacts, driven by water-intensive processes coupled with limited natural freshwater availability, and high water use in the electricity generation sector.

*Geographic Influence and SC Design*: Regions with water scarcity and inefficient water treatment, exhibit higher WD impacts. Scenarios with fabrication in water-abundant regions and advanced water recycling show significantly reduced WD.

*Material and Process Sensitivity*: Water use in substrate preparation, cleaning, and chemical synthesis is the major contributor to WD. Sapphire substrates used in InGaN production tend to consume more water compared to InGaP’s gallium arsenide substrates, explaining higher WD impacts for InGaN.

*Long-term Perspective (2050)*: WD impacts show less convergence than other impact categories due to local water availability constraints and differing regional water management practices. Sustainable water use improvements and circular water strategies will be critical to minimise long-term WD impacts, especially in water-stressed regions. The results of the other 14 impact categories are shown in the Supplementary Information file, Fig. S1.

### Phases contribution

**Fig. 3:**
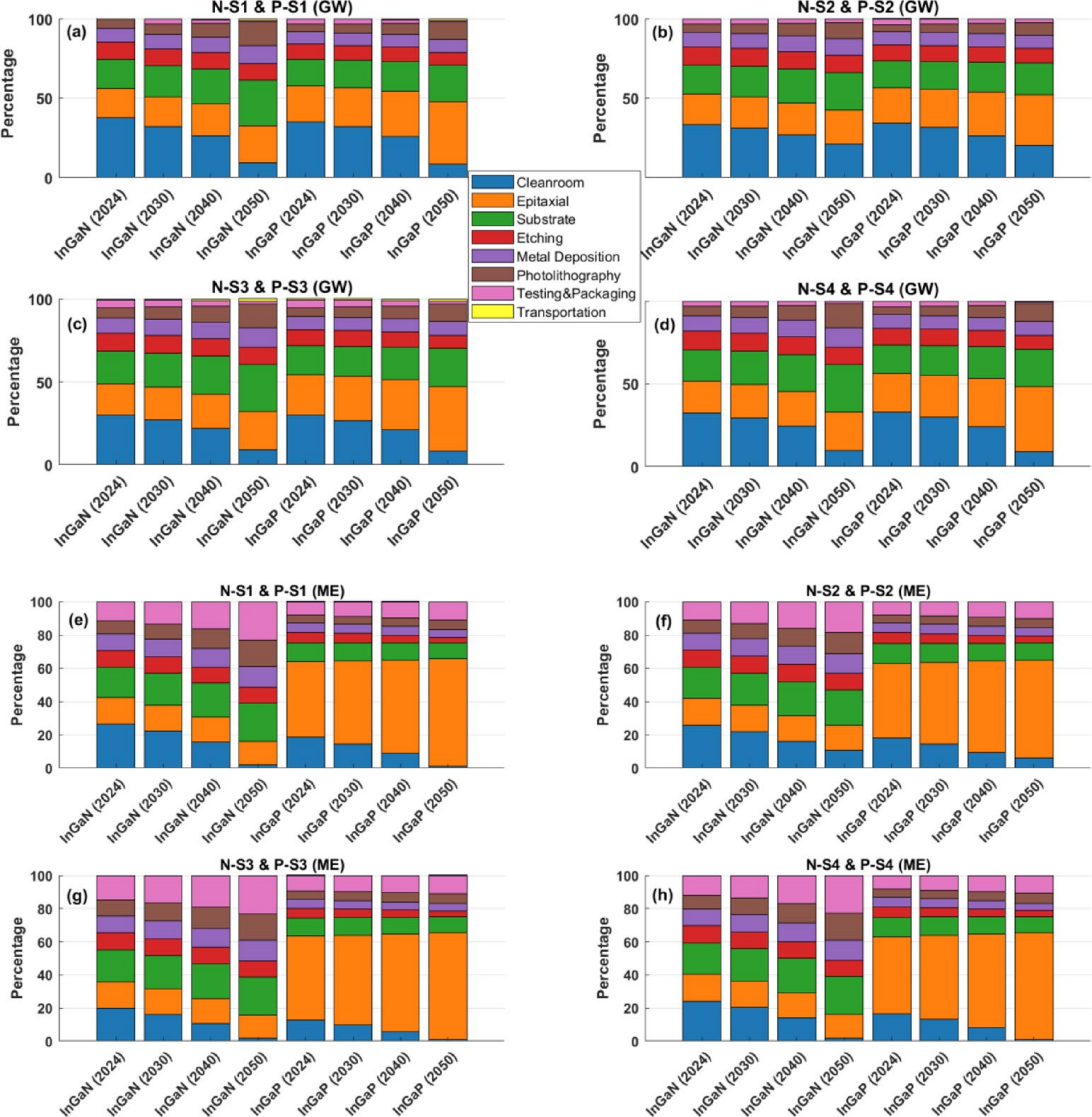

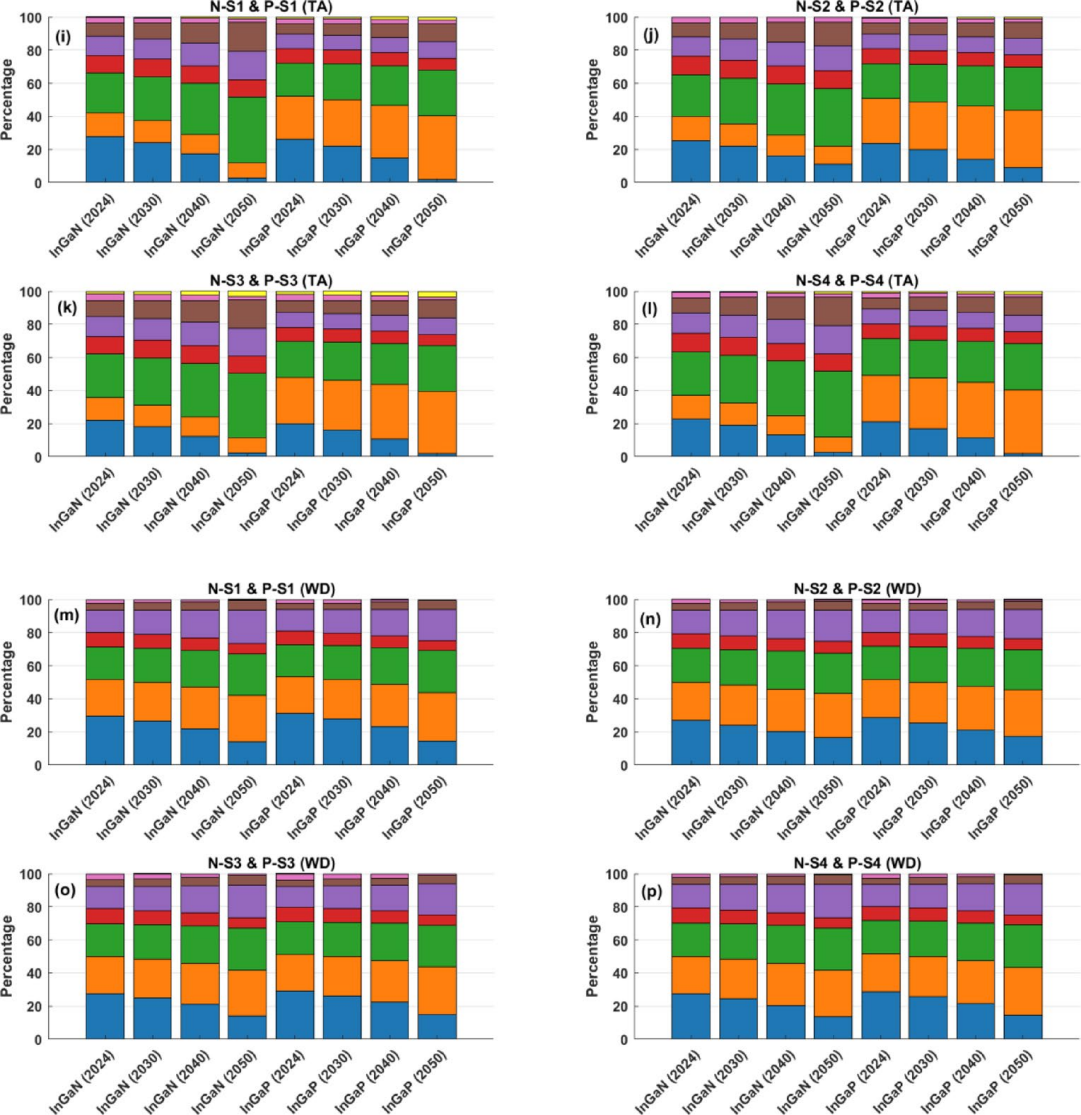
Contribution of each phase to the four main impact categories (GW, ME, TA, WD) across all four scenarios (N-S1 to N-S4 and P-S1 to P-S4) from 2024 to 2050 for InGaN and InGaP, (**a**–**d**) GW, (**e**–**h**) ME, (**i**–**l**) TA, (**m**–**p**) WD.

This geo-spatial temporal prospective LCA models the SC sustainability impact and identifies the phases contribution for the InGaN and InGaP SCs from 2024 to 2050 under a net-zero energy transition, assuming decarbonisation of electricity in considered countries. Figure 3 displays the contributions of the specific 8 phases in Scenarios N-S1 and P-S1to N-S4 and P-S4 across the four main impact categories (WP, ME, TA, WD).

Figure 3a,d illustrate the contribution of each phase to GW potential. For scenario N-S1, the cleanroom phase shows a significant drop in GW contribution - from 37.7 in 2024 to 9.3% in 2050 - highlighting the benefit of energy mix decarbonisation in electricity-intensive operations. In contrast, impacts from epitaxial growth, substrate preparation, and photolithography increase, with substrate rising from 18.3 to 28.9% and photolithography nearly tripling from 5.6 to 14.8%, indicating growing material and process intensity over time. This shows whilst electricity-related pollution decreases over time due to the development of renewables in the energy mix, material wafer growth and device-fabrication challenges intensified racing for bandgap breakthrough and nano meter scaling. In InGaP, a similar trend is observed: cleanroom impacts fall sharply (35.2–8.5%), while epitaxy becomes the dominant contributor by 2050, increasing from 22.5 to 39.4%. The photolithography shares also more than doubles, not because it becomes more energy-intensive, but because its material-driven impacts are less sensitive to grid decarbonisation than electricity-heavy steps like epitaxy and substrate preparation. Transportation and packaging remain minor contributors.

Under the net-zero energy scenario, ME impacts for InGaN and InGaP SCs shift notably between 2024 and 2050 (Fig. 3e,h). For InGaN, cleanroom impacts decline sharply from 26.4 to 1.9%, reflecting reduced emissions from electricity-intensive operations. However, testing and packaging increases markedly from 11.4 to 22.8%, becoming the largest contributor by 2050. Photolithography and metal deposition impacts also rise significantly (from 8.1 to 16.1%, and 9.8% to 12.3%, respectively), indicating increasing material and chemical use in advanced processes. In contrast, InGaP is consistently dominated by epitaxial growth, which rises from 45.3 in 2024 to 64.7% in 2050, making it the primary ecotoxicity hotspot. While cleanroom impacts reduce significantly (18.6–0.9%), other process contributions such as substrate preparation and metal deposition decline gradually. Testing and packaging show a moderate increase over time. Overall, while decarbonised electricity reduces ecotoxicity in energy-intensive phases, the growing impacts from epitaxy, photolithography, and testing and packaging suggest that future reductions must focus on cleaner materials and safer chemical handling in fabrication workflows.

Our prospective LCA results show distinct shifts in TA impacts across InGaN and InGaP SCs as electricity systems decarbonised (Fig. 3i,l). For InGaN, cleanroom-related impacts drop significantly from 27.9 in 2024 to 2.6% in 2050, while substrate preparation becomes the dominant contributor by 2050, increasing from 24.1 to 39.8%. Metal deposition and photolithography also rise steadily, from 11.4 to 17.1% and 8.1 to 17.4%, respectively, indicating higher acidifying emissions from material-intensive processes in future device manufacturing. In InGaP, epitaxial growth becomes the most critical contributor, growing from 26.0 to 38.3% by 2050. Similar to InGaN, cleanroom impacts fall sharply (26.1–2.0%), and substrate impacts rise. Etching, and testing and packaging impacts gradually decline, while photolithography increases to 10.9%, reinforcing its growing environmental relevance. These results highlight that while clean energy adoption reduces acidification from energy-intensive phases, future impacts are increasingly driven by material sourcing and chemically intensive fabrication. Addressing acidification risks will require cleaner upstream SCs and alternative sustainable deposition and chemistries.

Under the Net-Zero energy transition scenario, the WD impacts in both InGaN and InGaP SCs are presented in Fig. 3m,p. For InGaN, the cleanroom contribution declines from 29.6 in 2024 to 13.9% in 2050, reflecting reductions from electricity-related water use. However, epitaxial growth and substrate preparation increase steadily, together accounting for over 53% of total impact by 2050. Metal deposition also rises significantly (from 13.5 to 20.1%), emphasising water-intensive material processing in future InGaN manufacturing. Similarly, for InGaP, cleanroom impacts fall from 31.1 to 14.6%, while epitaxy becomes the largest contributor by 2050, growing from 22.2 to 29.1%. Substrate and metal deposition phases follow similar increasing trends. In both materials, etching, photolithography, and testing and packaging decline in relative contribution, suggesting limited water savings potential in those phases. In all scenarios, the relative contribution from the transportation phase is negligible. The results of the other scenarios are presented in the Supplementary Information file, Fig. S2.

### Sustainability scoring

**Fig. 4:**
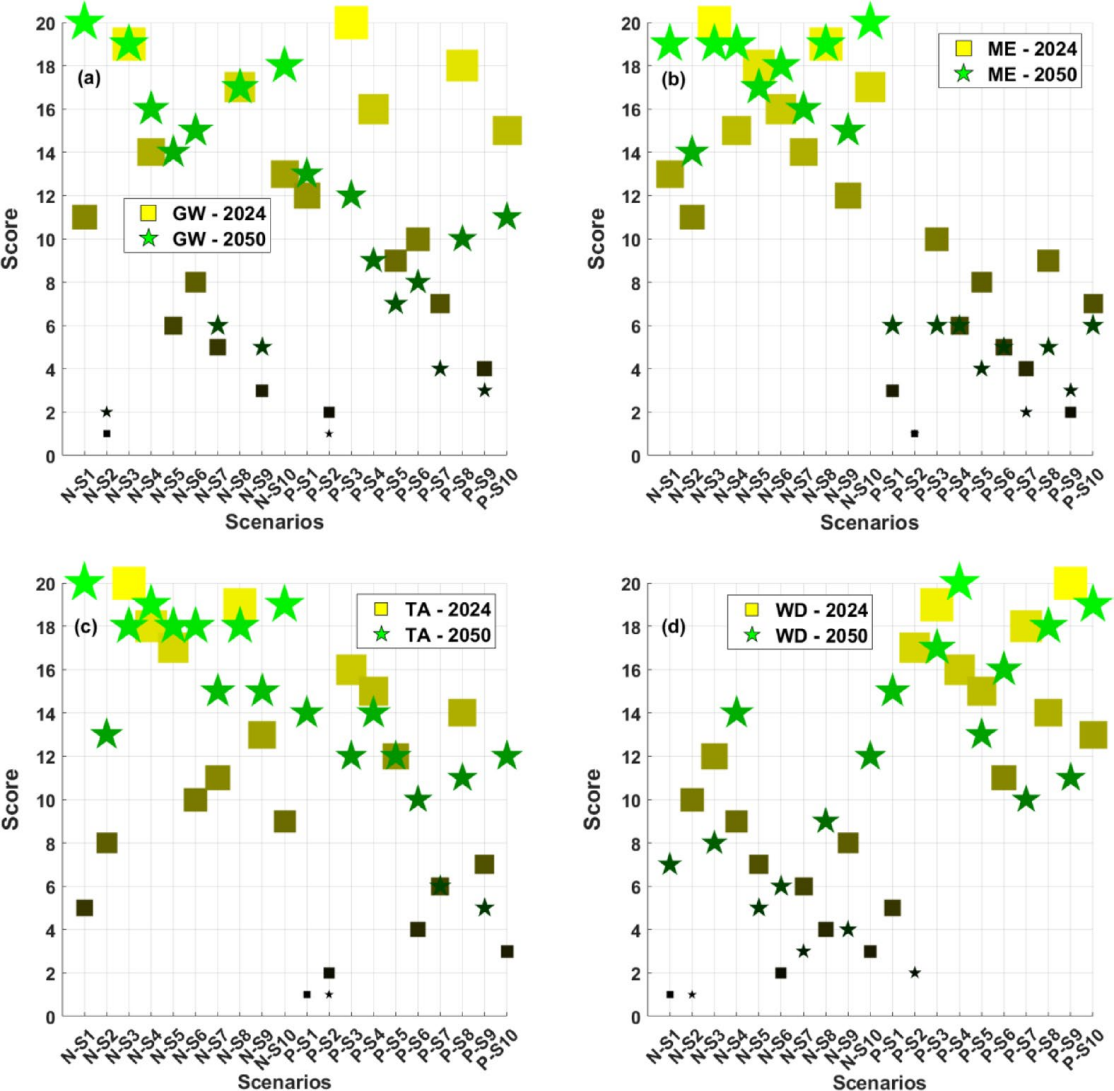
Sustainability rankings of 80 international supply chain scenarios for InGaN and InGaP compound semiconductors across four impact categories for 2024 and 2050. (**a**) GW, (**b**) ME, (**c**) TA, and (**d**) WD.

Between 2024 and 2050, the scenario-based LCA results reveal pronounced variability in sustainability performance by impact categories across 80 distinct international SC configurations for InGaN and InGaP compound semiconductors. Each scenario is ranked from 1 (least sustainable) to 20 (most sustainable) according to the results obtained from the geo-spatial prospective LCA modelling. The sustainability rankings for the four impact categories for both technologies are shown in Figs. 4a–d.

Based on GW impact, scenarios N-S3 emerge as top performers in both 2024 and 2050, with a consistent rank of 19. Also, N-P3 holds the highest rank in 2024. These scenarios feature fabrication in the UK, testing and packaging in Malaysia and use-phase in Japan, suggesting that leveraging low-carbon energy mixes, efficient logistics, and regional integration significantly reduce life cycle greenhouse gas emissions. In addition, N-S1 shows strong potential to improve its rank from 11 to 20 between 2024 and 2050. In contrast, N-S2 and P-S2, despite being regionally consolidated in China, score 1–2 in 2024 and remain at the low end in 2050, indicating that China’s high process emissions and energy intensity continue to dominate GW impact, even under projected decarbonisation scenarios (Fig. 4a).

ME scores show broader dispersion. P-S2, P-S9, and P-S1 have the lowest scores in 2024 (1, 2, and 3, respectively), which indicates a higher environmental burden. In contrast, N-S3 and N-S8 score 20 and 19, indicating strong sustainability in ME due to more responsible metal sourcing and lower material intensity in those regions. By 2050, P-S2 remains low at 1, showing little improvement (Fig. 4b), indicating localisation of materials sourcing, manufacturing, testing and packaging, and use phases largely in a single country do not necessarily lead to the most sustainable SC.

TA results are highly sensitive to regional air pollution profiles (e.g., SO₂, NOx emissions from electricity generation). In 2024, N-S3 scores 20 and P-S2 scores 2, again showing that N-S3’s UK-based fabrication helps avoid acidifying emissions, while P-S2, centred in China, performs poorly. In 2050, TA scores improve across scenarios, but P-S2 remains at 1, showing that regional electricity and industrial emissions in China still pose challenges even with assumed grid improvements. Scenarios like N-S1, N-S2, N-S6, N-S10, P-S1, P-S6, and P-S10 continue to show significant TA improvement, benefiting from more favourable energy mixes or geographic dispersion (Fig. 4c).

WD scores reveal significant differences across scenarios and years. In 2024, N-S1, N-S6, and N-S10 show the worst performance (scores of 1–3), likely due to high water consumption for electricity generation or operations in water-stressed regions. In contrast, P-S9, and P-S3 achieve the highest scores (19–20), indicating more water-efficient processes or favourable local water availability. By 2050, some scenarios like N-S2 and P-S2 drop in their WP scores, suggesting increasing regional water stress or process intensification. Since China’s net zero target is 2060, it means that in 2050 this country will still be using coal, natural gas, and oil-based electricity generation in its energy mix, which causes higher WD impact compared to other scenarios. These variations underscore the importance of incorporating long-term water sustainability into SC planning (Fig. 4d). The ranking results of the other 14 impact categories are shown in the Supplementary Information file, Fig. S3.

By focusing on 2024 and 2050, Table 2 effectively highlights the improvement from the current state to the long-term target, emphasising the potential benefits of decarbonisation, and cleaner energy over the full prospective horizon. This table summarises the best and worst scenarios based on the geo-spatial prospective LCA results for 2024 and 2050 for all 18 impact categories. Based on the LCA impact categories, the most sustainable scenarios are consistently found among the N-S3 and N-S10 configurations across most categories. Specifically, N-S3 emerges as the most sustainable scenario, appearing as the best case in 10 out of 18 categories, including critical ones like:

- Fine particulate matter formation.
- Freshwater ecotoxicity.
- Human toxicity (both carcinogenic and non-carcinogenic).
- Land use.
- Marine eutrophication.
- Ozone formation.
- Terrestrial ecotoxicity.

N-S10 is also favourable in the 2050 outlook for categories such as:

- Marine ecotoxicity.
- Mineral resource scarcity.
- Marine eutrophication.
- Human toxicity.

Meanwhile, the most polluting (worst-case) scenarios are largely associated with P-S2, which appears as the worst scenario in 11 out of 18 categories, especially in:

- Global warming.
- Freshwater and marine toxicity.
- Ozone formation.
- Terrestrial and human toxicity.
- Ionising radiation.

Other high-impact worst-case contributors include P-S1, P-S4, and N-S2/N-S4 (e.g., N-S2 in GW, N-S4 in stratospheric ozone depletion). A detailed narrative and full breakdown of scenario results are provided in the Supplementary Information.

**Table 2:** Summary of best and worst scenarios in 2024 and 2050 for all 18 impact categories.

Impact category	Best 2024	Best 2050	Worst 2024	Worst 2050
Fine particulate matter formation	N-S3	N-S1	P-S1	P-S2
Fossil resource scarcity	P-S3	N-S3	N-S4	P-S2
Freshwater ecotoxicity	N-S3	N-S10	P-S2	P-S2
Freshwater eutrophication	N-S3	N-S1	P-S2	P-S2
Global warming	P-S3	N-S1	N-S2	P-S2
Human carcinogenic toxicity	N-S3	N-S1	P-S2	P-S2
Human non-carcinogenic toxicity	N-S3	N-S10	P-S2	P-S2
Ionising radiation	N-S3	N-S4	P-S2	P-S2
Land use	N-S3	N-S10	P-S2	P-S2
Marine ecotoxicity	N-S3	N-S10	P-S2	P-S2
Marine eutrophication	N-S3	N-S10	P-S2	P-S2
Mineral resource scarcity	N-S3	N-S10	P-S4	P-S2
Ozone formation, Human health	N-S3	N-S3	P-S2	P-S2
Ozone formation, Terrestrial ecosystems	N-S3	N-S3	P-S2	P-S2
Stratospheric ozone depletion	P-S3	P-S1	N-S4	N-S2
Terrestrial acidification	N-S3	N-S1	P-S1	P-S2
Terrestrial ecotoxicity	N-S3	N-S10	P-S9	P-S9
Water Depletion	P-S9	P-S4	N-S1	N-S2

### Discussions and conclusions

This prospective scenario-based LCA of InGaN and InGaP compound semiconductor technologies reveals significant temporal and spatial variability in environmental impacts, with clear implications for sustainable SC design. Across 80 international SC configurations demonstrate marked reductions in environmental impacts from 2024 to 2050, primarily due to global electricity grid decarbonisation, and improved emissions controls.

#### Temporal trends and decarbonisation effects

Across all impact categories, cleaner electricity grids emerge as the primary driver of impact reduction. These reductions reflect the projected decarbonisation of electricity not only in fabrication regions but also across upstream material extraction and processing stages, ensuring that the benefits of cleaner energy are propagated throughout the entire SC. For instance, InGaN’s GW impact in Scenario N-S1 (Taiwan fabrication) drops by 76% from 1.77 to 0.42 kg CO₂ eq/cm², while ME, TA, and WD show parallel declines. By 2050, scenarios converge toward similar environmental baselines, reflecting the global push toward net-zero energy, although regional disparities persist.

#### Scenario and geographic influence

Scenarios with fabrication in low-carbon regions (e.g., UK-based Scenarios N-S3 and P-S3) consistently outperform others across most environmental metrics. In contrast, configurations centred in coal-reliant countries such as China (e.g., Scenarios N-S2 and P-S2) exhibit the highest impacts, even in 2050. These differences highlight the enduring influence of regional electricity profiles, pollution control practices, and water resource management. Notably, even under future decarbonisation assumptions, China-centred scenarios remain environmental hotspots in the SC across GW, TA, ME, and WD categories.

#### Process-level contributions and shifting impact hotspots

While electricity-intensive cleanroom processes show steep declines in contribution over time (e.g., from ~ 35% to < 10%), impacts from epitaxial growth, substrate preparation, photolithography, and packaging increase in relative importance. For instance, epitaxial growth becomes the dominant contributor to ME and TA in InGaP by 2050 (64.7% and 38.3%, respectively), underscoring the rising environmental burden of material- and chemical-intensive phases. These findings stress the growing need for process innovation, cleaner precursors (e.g., replacing arsine or phosphine), and advanced material recycling.

#### Technology-specific trends

InGaN generally performs better than InGaP in most categories, attributed to its simpler material inputs and lower toxicity potential. InGaP scenarios exhibit higher ME, carcinogenic toxicity, and mineral resource scarcity, driven by complex chemistries and GaAs substrates. However, InGaP scenarios significantly outperform InGaN in stratospheric ozone depletion due to limited use of halogenated chemicals. This trade-off reinforces the need for application-specific decision-making in technology deployment.

#### Hotspot scenarios and sustainability leaders

Across all years and impact categories, Scenario N-S3 (UK fabrication, Malaysia testing and packaging) emerges as the most environmentally sustainable pathway, consistently scoring among the highest in GW, ME, TA, Fine Particulate Matter Formation (FPMF), Fossil Resource Scarcity (FRS), Freshwater Eutrophication (FEU), and toxicity-related categories. Conversely, Scenario 2 (China-based) remains a persistent outlier with the poorest sustainability performance. High-performing InGaN scenarios (e.g., N-S3, N-S8) demonstrate the value of low-carbon grids, responsible material sourcing, and regional integration, whereas InGaP pathways (e.g., P-S2, P-S9) suffer from persistent chemical and resource-related burdens.

#### Reshoring implications

The scenario analysis also provides compelling evidence to support reshoring or nearshoring of compound semiconductor fabrication to regions with cleaner energy profiles and stronger environmental regulations. Scenarios involving the UK, USA and Taiwan (specially in 2050), consistently achieve higher sustainability scores across GW, toxicity, and resource depletion categories. These findings suggest that strategic reshoring - particularly of energy- and material-intensive phases such as epitaxy and substrate preparation - can substantially reduce life cycle environmental burdens. Beyond emissions reduction, reshoring can enhance SC resilience and align with emerging national policies aimed at sustainable and secure semiconductor production.

#### Limitations

This study applies a scenario-based prospective approach in which electricity-mix decarbonisation is treated as the primary driver of temporal change. Other factors (such as potential future process innovations, changes in tool efficiency, material substitutions, or shifts in production yields) are held constant to isolate the influence of energy-system transitions. As a result, the projected trajectories should be interpreted as illustrative rather than predictive. Specific Bills of Materials (BOMs) and facility-level process parameters cannot be disclosed due to commercial confidentiality; instead, national-average inventories compiled from multiple facilities are used. These methodological choices enhance transparency and comparability but limit the ability of the study to forecast technology-specific operational performance or to capture detailed facility-level heterogeneity.

#### Policy and research implications

The findings highlight the urgent need for targeted policies that decouple compound semiconductor fabrication from high-impact energy and material sources. Effective strategies include prioritising clean grid access for energy-intensive processes, mandating safer precursor chemicals, enhancing global wastewater treatment standards, and incentivising circular economy solutions for water, materials, and rare metals. Furthermore, regional and global SC optimisation must integrate long-term environmental forecasts into design choices to ensure resilient and sustainable semiconductor manufacturing.

Based on the results of this study, the following recommendations are proposed to enhance the sustainability of compound semiconductor SCs:

- Prioritising low-carbon energy regions for fabrication phases to reduce electricity-related impacts.
- Reshoring or nearshoring key fabrication phases to environmentally efficient regions with strong regulatory frameworks.
- Investing in cleaner and safer precursor chemicals to minimise toxicity and occupational hazards.
- Enhancing process-level energy and material efficiency through advanced reactor and tool design.
- Advancing water recycling and process water management systems to mitigate water depletion impacts.
- Developing circular economy strategies for recovering and reusing critical raw materials like indium and gallium.
- Supporting global harmonisation of environmental standards and emissions control practices.

## Methods

### Life cycle assessment

Over the past three decades, LCA has evolved into a key methodology for environmental management and decision support, providing a scientific basis for the development of policies and strategies^45–47^. LCA is a widely used and effective method for identifying the environmental impacts of various processes, products, and systems^48^. Its application helps tackle critical sustainability challenges, supporting future development and growth^49,50^. LCA is a powerful tool for guiding informed decision-making by systematically evaluating various environmental impacts across the entire product development value chain^51,52^. LCA is widely regarded as a reference methodology for providing decision support within policy contexts^53^. The fundamental principle of LCA involves tracing a product throughout its life cycle and establishing a boundary between its product system and the external environment. The energy and material flows that cross this boundary are linked to the system’s inputs^54^. The LCA framework for a product, process, or activity can encompass the combined impacts of collaborative SC partners, including raw material extraction and processing, manufacturing, transportation, and distribution, as well as reuse, maintenance, recycling, and final disposal^2^. The insights derived from LCAs must be translated into actionable policies, including taxation measures, regulatory frameworks, and incentive mechanisms. Additionally, these findings should inform the development of innovative business models and provide guidance for consumers and investors^55^. Based on this foundation, this study integrates geo-spatial analytics with traditional process-based LCA method to model the prospective environmental sustainability of InGaN and InGaP compound semiconductor SCs. A total 11 countries with geographic regions spanning USA, UK, Germany, China, Taiwan, Malaysia, South Korea, Japan, Canada, Morrocco and Russia are considered across 4-time horizons—2024 (baseline), 2030, 2040 and 2050. A total of 80 international InGaN and InGaP compound semiconductor SCs scenarios are modelled.

1. Goal and scope definition.

The core element of LCA research is the establishment of evaluation objectives. This phase aims to establish a defined boundary for evaluating environmental impacts, addressing the vast array of parameters that could potentially be analysed within a production chain^9^. The objective of an LCA study should clearly define its intended application, the purpose of the analysis, and the target audience for the results. This ensures clarity regarding the data and units to be included in the study^9,56^. Defining functional units and system boundaries in relation to these objectives shapes the structure of the analysis, ultimately enabling the assessment of various environmental impact categories^57^. This phase entails specifying the LCA type to be applied, articulating the study’s objective, and determining the system boundaries^58^.

The main objective of this research is to conduct a comparative assessment of the SC environmental impact throughout the life cycle of two compound semiconductor technologies: InGaN and GaInP. This environmental impact comparison is conducted based on international SC scenarios comprising leading countries involved in critical material supply, technologies fabrication, testing and packaging and usage phases. Conducting prospective LCA based on varying energy mix in countries involved in developed SC scenarios following the net-zero target by 2050 is another main goal of this study. Temporal projections for 2030, 2040, and 2050 were based on increasing shares of renewable electricity in the national energy mixes of all countries included in the SC models. This decarbonisation affects not only the electricity consumed during fabrication, testing, and packaging processes but also the energy embedded in upstream stages such as raw material extraction, precursor production, and substrate processing. Process efficiencies were assumed constant over time, allowing the analysis to isolate the effect of energy transition on overall SC environmental impacts. In line with the relevant ISO 14,044 standard, any product system within an LCA must be defined by a function that represents the performance characteristics of the system^49^. Functional units provide a consistent basis for comparing resource use and emissions in life cycle assessments^59^. The considered functional unit in this study is per 1 cm^2^ of processed wafer which means that the study’s analysis and calculations are based on a reference unit of 1 cm² of a semiconductor wafer that has undergone all fabrication processes from material supply to use. All data, impacts, and results are evaluated relative to this standardised unit to allow for consistent comparisons and assessments. This choice of functional unit reflects industrial fabrication practice and is particularly relevant for analysing manufacturing-scale challenges related to material supply, energy demand, and high-technology processing in compound semiconductor supply chains. It also enables a technology-neutral comparison of manufacturing-related environmental burdens across InGaN and InGaP devices. Performance-related parameters such as external quantum efficiency (EQE) and use-phase electricity consumption are outside the system boundary of this study and are therefore not reflected in the area-based functional unit. The considered system boundary in this study is cradle-to-gate which means raw material extraction, transportation and distribution, fabrication, test and packaging and use phases are included in this study.

2. Data inventory.

Data acquisition represents a critical step in the LCA process^60,61^. This phase is the most challenging and time-consuming stage in an LCA^62^. In LCA, potential environmental impacts throughout a product or service’s life cycle are evaluated using a life cycle inventory (LCI), which comprises relevant input/output data and emissions associated with the system under analysis^63^. Unavailable data must be supplemented with estimations, secondary, and generic data, which can introduce higher levels of uncertainty and constrain the study’s scope^62^. The inventory analysis involves compiling, qualifying, and quantifying inputs and outputs, represented through resources, materials, and emissions within the examined SCs^64^. Both primary and secondary data are used to create the LCI for the system modelling. Primary data collected from industry partners, such as exact material quantities, energy use, and specific process parameters, are confidential and cannot be disclosed.

To enhance transparency, the overall data composition of the life cycle inventory is approximately as follows: ~15% derived from Ecoinvent v3.9 database processes (including electricity generation, material production, fabrication-related background processes, and transportation), ~ 10% from peer-reviewed literature sources, ~ 75% from industry reports and technical documentation, and aggregated confidential communications and industrial insights. All background and fabrication-related processes were modelled using Ecoinvent v3.9 datasets to ensure methodological consistency across the system boundary. All SC data represent average values across multiple companies and facilities within each country, providing a representative national-level perspective rather than data from any individual manufacturer. This approach ensures results reflect typical industry practices, enhancing generalizability and robustness.

Due to commercial confidentiality, specific BOMs and proprietary process details cannot be disclosed. However, the data used in this study represent national averages across multiple facilities, reflecting typical regional manufacturing practices. Each fabrication process may, in practice, consume slightly more or less material, water, or chemicals than the average. These variations generally offset each other, and minor fluctuations in energy, material inputs, or emissions are inherently captured within the averaged national datasets. Internal consistency checks and qualitative sensitivity considerations indicate that typical variations of approximately ± 10% in key inputs would not meaningfully affect the overall LCA outcomes. A supplementary table (Table S1) presents the variation in GW impacts resulting from + 10% changes in key input parameters, including energy, gaseous precursors, and chemicals. This demonstrates that typical fluctuations in material and energy inputs lead to only minor variations in overall environmental impacts. Therefore, the results are robust, representative of real-world manufacturing variability, and provide a reliable depiction of SC environmental performance without compromising confidential information.

Transportation impacts were calculated per functional unit, covering the full supply chain: transport of raw materials to fabrication facilities, fabricated wafers to testing and packaging sites, and finished devices to use-phase locations. Distances were estimated using Google Maps^65^ for representative country-to-country routes, and emission factors for road, sea, and air freight were taken from Ecoinvent 3.9. Impacts were allocated according to the mass of materials and devices transported and normalized to the functional unit. Despite intercontinental air freight in some scenarios, transportation contributes less than 1% to total environmental impacts compared to the energy- and material-intensive fabrication processes.

3. Life cycle impact assessment methodology.

According to ISO 14,044 standard, the Life Cycle Impact Assessment (LCIA) framework comprises mandatory elements, such as characterisation, optional components normalisation and weighting. The selection of impact categories is determined by the authors’ discretion and is guided by data availability and the study’s specific objectives^64^. The LCIA stage aims to evaluate the environmental impacts and analyse the data to assess the contribution of each impact category, based on the inventory analysis, within the context of the goal and scope defined in the study^66^. LCIA evaluates potential environmental impacts by translating LCI data into specific impact indicators^8^. This stage includes the processes of classification, characterisation, normalisation, evaluation, and weighting of the data, based on the impact categories defined in the study^62^. Various methods are available for calculating LCIA results^8^. The ReCiPe 2016 midpoint (H) has been selected to calculate 18 impact categories.

4. Results interpretation.

Through the three steps outlined above, the research analysis subject is identified, system boundaries for the inventory are defined, and model analysis is incorporated to evaluate environmental burdens during the impact assessment. This process offers actionable recommendations, facilitating adjustments to system variables and addressing the root causes of environmental impacts^57^. Interpretation involves analysing the outcomes of the inventory analysis and impact assessment to identify significant environmental impacts. This phase also includes evaluating the results for completeness, sensitivity, and consistency to ensure their reliability and relevance^64^. The interpretation phase is crucial as it ensures that the impact assessment results are both comparable and comprehensible^9^.

5. Geo-spatial supply chain and prospective LCA integration.

Decision making on sustainability is incomplete if the SC is excluded in the systems boundary. Environmental impact varies based on geography and time. Integrating LCA into SC management offers significant benefits^67^. Integrating the LCA approach with SC scenario analysis establishes a synergistic framework for advancing sustainability. This combined approach merges the thorough assessment of environmental impacts across a product’s life cycle with the strategic implementation of sustainable practices throughout the SC ^68^. The integrated approach helps identify key areas where environmental impacts can be reduced beyond the manufacturing processes such as improving energy efficiency during wafer processing, minimising material waste, or enhancing recycling practices, but also incorporating upstream sustainable sourcing of materials and downstream sustainable use. Additionally, scenario analysis allows for the evaluation of different conditions, such as alternative materials, energy sources, or recycling practices, to determine which strategies lead to the best environmental outcomes^68^. A frequent challenge in conducting LCA is the availability and reliability of the required data. Acquiring precise information on inputs, processes, and environmental impacts throughout the entire product life cycle can be both complex and resource-demanding^68^.

The integrated geo-spatial SC LCA provides quantitative prospective insights, enabling stakeholders to make and adjust informed choices that prioritise sustainability across manufacturing and SC over future time horizons, while allowing businesses to communicate their sustainability efforts to stakeholders, including customers, regulators, and investors^68^. This integration empowers organisations to embed geo-spatial-driven decision-making, prioritise sustainability efforts, and drive continuous improvements in environmental performance. Building from this integration, a total of 80 international SC scenarios for InGaN and InGaP across 2024, 2030, 2040 and 2050 are designed and modelled. For the prospective LCA scenarios (2030, 2040, 2050), we adjusted the electricity mix of each country involved in the SCs by gradually increasing the share of renewable energy relative to the 2024 baseline from International Energy Agency (IEA) data. This scenario-based approach enables the assessment of temporal trends in environmental impacts under decarbonising energy systems. All other process inputs were kept constant to isolate the influence of electricity decarbonisation. This method provides a transparent illustration of trends toward net-zero targets without relying on statistical forecasting or complex predictive models.

While future technological improvements (such as enhanced precursor utilisation, increased water recycling, reduced epitaxy temperatures, improved yields, and other process optimisations) could influence the environmental performance of InGaN and InGaP devices, these developments are inherently uncertain and difficult to predict. All process parameters are held constant from 2024 to 2050, allowing this study to isolate the effect of electricity decarbonisation on prospective environmental impacts.

6. Scenario-based sustainability ranking.

A sustainability scoring and ranking framework is developed to enable a structured and comparative evaluation of the environmental performance of all international SC configurations. Each scenario is assessed across 18 ReCiPe 2016 midpoint impact categories for both 2024 and 2050, reflecting current and projected environmental conditions against the net-zero goals.

A discrete scoring scale from 1 to 20 is applied to each impact category, where 1 indicates the scenario with the poorest environmental performance (i.e., highest impact) and 20 represents the most sustainable scenario (i.e., lowest impact). This scoring approach captures the relative performance of each scenario in individual impact categories and enables comparison across a wide range of environmental indicators.

The overall sustainability performance of each scenario in a given year is then determined by summing its scores across all impact categories. This method provides a transparent and reproducible way to identify top- and bottom- performing scenarios under both present-day and future conditions, accounting for changes in technology, energy systems, and regional characteristics.

## Supplementary Information

Below is the link to the electronic supplementary material.

Supplementary Material 1

## Data Availability

The datasets generated and/or analysed during the current study are not publicly available due to confidentiality matters but are available from the corresponding author on reasonable request.

## Abbreviations

As: Arsenic
BOM: Bills of material
EQE: External quantum efficiencies
FE: Freshwater ecotoxicity
FEU: Freshwater eutrophication
FPMF: Fine particulate matter formation
FRS: Fossil resource scarcity
Ga: Gallium
GaAs: Gallium arsenide
Ge: Germanium
GW: Global Warming
HCT: Human carcinogenic toxicity
HNCT: Human non-carcinogenic toxicity
IEA: International energy agency
In: Indium
InGaN: Indium gallium nitride
InGaP: Indium gallium phosphide
IR: Ionising radiation
LCA: Life cycle assessment
LCI: Life cycle inventory
LCIA: Life cycle impact assessment
LED: Light-emitting diode
LU: Land use
ME: Marine ecotoxicity
MEU: Marine eutrophication
MRS: Mineral resource scarcity
MOCVD: Metal-organic chemical vapor deposition
OFHH: Ozone formation, human health
OFTE: Ozone formation, Terrestrial ecosystems
PFC: Perfluorocarbons
QCSE: Quantum-confined Stark effect
QW: Quantum well
RGB: Red-green-blue
SC: Supply chain
SOD: Stratospheric ozone depletion
TA: Terrestrial acidification
TE: Terrestrial ecotoxicity
TMAl: Trimethylaluminum
TMGa: Trimethylgallium
TMIn: Trimethylindium
WD: Water depletion
WBG: Wide band gap
