# Supplementary material for: Geo-spatial prospective life cycle sustainability of InGaN and InGaP compound semiconductors
Source: Sci Rep. 2026 Mar 16;16:13659. doi: 10.1038/s41598-026-43622-5 (PMC13125600; doi:10.1038/s41598-026-43622-5)
Supplement: Supplementary file 1 — Supplementary Material 1 [file 41598_2026_43622_MOESM1_ESM.pdf]

Supplementary Information (SI)

Geo-spatial prospective life cycle sustainability of InGaN and InGaP compound semiconductors

Moein Shamoushaki [a,b]*, Josie Travers-Nabialek [c], Sara-Jayne Gillgrass [c], Peter M. Smowton [c], S.C. Lenny Koh [a,b]*

[a] Sheffield University Management School, The University of Sheffield, Sheffield, S10 1FL, United Kingdom

[b] Energy Institute, The University of Sheffield, Sheffield, S10 2TN, United Kingdom

[c] School of Physics and Astronomy, Cardiff University, Cardiff, CF24 3AA, United Kingdom

* Corresponding authors: m.shamoushaki@sheffield.ac.uk , s.c.l.koh@sheffield.ac.uk

Supplementary information contents

Headings:

Geo-spatial prospective LCA results

Environmental impact breakdown by process

Sustainability performance rankings

Figures:

Fig S1. Projected environmental impact for InGaN and InGaP semiconductors across 14 categories from 2024 to 2050 comparing FPMF, FRS, FE, FEU, HCT, HNCT, IR, LU, TE, MEU, MRS, OFHH, OFTE, SOD results from the 80 international supply chain scenarios

Fig S2. Impact contributions from 8 processes for InGaN and InGaP compound semiconductors for scenarios N-S5 to N-S10 and P-S5 to P-S10 comparing the WP, ME, TA and WD results between InGAN and InGaP from 2024 to 2050

Fig S3. Sustainability ranking results by 14 indicators (FPMF, FRS, FE, FEU, HCT, HNCT, IR, LU, MEU, MRS, OFHH, OFTE, SOD, TE) for InGaN and InGaP across all international supply chain scenarios in 2024 and 2050

Table:

Table S1. Sensitivity of Global Warming impacts to +10% variations in key input parameters

Geo-spatial prospective LCA results

Fig S1 show the geo-spatial prospective LCA results for InGaN and InGaP compound semiconductors across 14 impact categories for 2024, 2030, 2040 and 2050 totalling 80 international supply chain scenarios. Both InGaN and InGaP exhibit consistent environmental impact reductions across all assessed categories from 2024 to 2050, with InGaN generally performing slightly better. The Fine Particulate Matter Formation (FPMF) category shows steady decreases, especially in Scenarios N-S3, P-S3, N-S4 and P-S4, driven by cleaner electricity and more integrated processes. Despite InGaN's higher-temperature operations, its simpler process inputs contribute to lower impacts compared to InGaP. Major hotspots include epitaxial growth and the use of metalorganic precursors like TMIn and TMGa. Similar trends are seen in Fossil Resource Scarcity (FRS), with both materials achieving over 70% reductions by 2050 in most scenarios. InGaN's reliance on fossil-derived hydrogen and TMIn results in higher impacts, while N-S3 and P-S3 achieve the lowest fossil depletion values. In contrast, N-S4 and P-S4 lag due to slower decarbonisation. Freshwater Ecotoxicity (FE) and Eutrophication (FEU) impacts also decline significantly, particularly in InGaN scenarios, as cleaner upstream production, and more sustainable chemical sourcing come into effect. InGaP tends to show higher ecotoxicity due to its more complex material inputs and greater potential for metal leaching. Scenario 3 remains the best performer, with InGaN Scenario 1 showing a 60% ecotoxicity reduction, and eutrophication potential decreasing by up to 84%.

Human health-related impacts follow a similar downward trend. Carcinogenic toxicity improves across all scenarios, with InGaP maintaining slightly higher values. N-S1, for example, reduces carcinogenic impact by 56% by 2050, with Scenario 3 again showing the lowest overall toxicity for both semiconductors. Human non-carcinogenic toxicity (HNCT) reductions are more pronounced for InGaN, which starts with lower baseline values and shows steeper declines. By 2050, several InGaN scenarios converge around 0.366 kg 1,4-DCB/cm^2, while InGaP values remain up to 2.5 times higher due to its chemically intensive processes. N-S3 and P-S3 demonstrate the most consistent improvements, highlighting the benefits of low-toxicity precursors and better emissions controls. Ionising radiation (IR) impacts decrease more modestly, mainly driven by cleaner energy mixes. Scenarios N-S3 and P-S3 again performs best, though all scenarios gradually converge by 2050, suggesting that improvements in power generation are more impactful than process changes. Land use impacts also show steady reductions, especially for InGaN, which benefits from more land-efficient energy sources. Scenario N-S3 achieves a 44% reduction, while P-S3 sees up to a 28% drop in its best scenario. Marine eutrophication (MEU) also improves, with InGaN Scenario N-S3 showing a 30% decrease and InGaP improving by 24%, driven by upstream nutrient emissions control.

Other impact categories exhibit less variability but still show improvement. Mineral Resource Scarcity (MRS) decreases only slightly, reflecting persistent reliance on critical raw materials (InGaN shows a modest 9% reduction. Ozone Formation impacts (both for human health and terrestrial ecosystems) decline meaningfully over time. InGaN sees a 60.7% drop in Scenario N-S1 for human health ozone impacts, while InGaP improves by 43.8% in Scenario P-S3. Terrestrial ozone impacts also fall steadily, with InGaN and InGaP both benefiting from cleaner upstream processes. Stratospheric ozone depletion (SOD) shows only marginal improvement, as the semiconductor processes release minimal ozone-depleting substances; however, N-S3 and P-S3 still perform best due to cleaner supply chains. Lastly, Terrestrial Ecotoxicity (TE) shows moderate reductions, with InGaN achieving a 14.6% drop in Scenario N-S3 and InGaP improving

by only about 4%, again reflecting differences in materials and emissions profiles. Overall, N-S3 and P-S3 consistently deliver the lowest environmental impacts across nearly all categories, underscoring the importance of decarbonised energy, efficient material use, and cleaner supply chains in minimising the environmental burdens of compound semiconductor manufacturing.

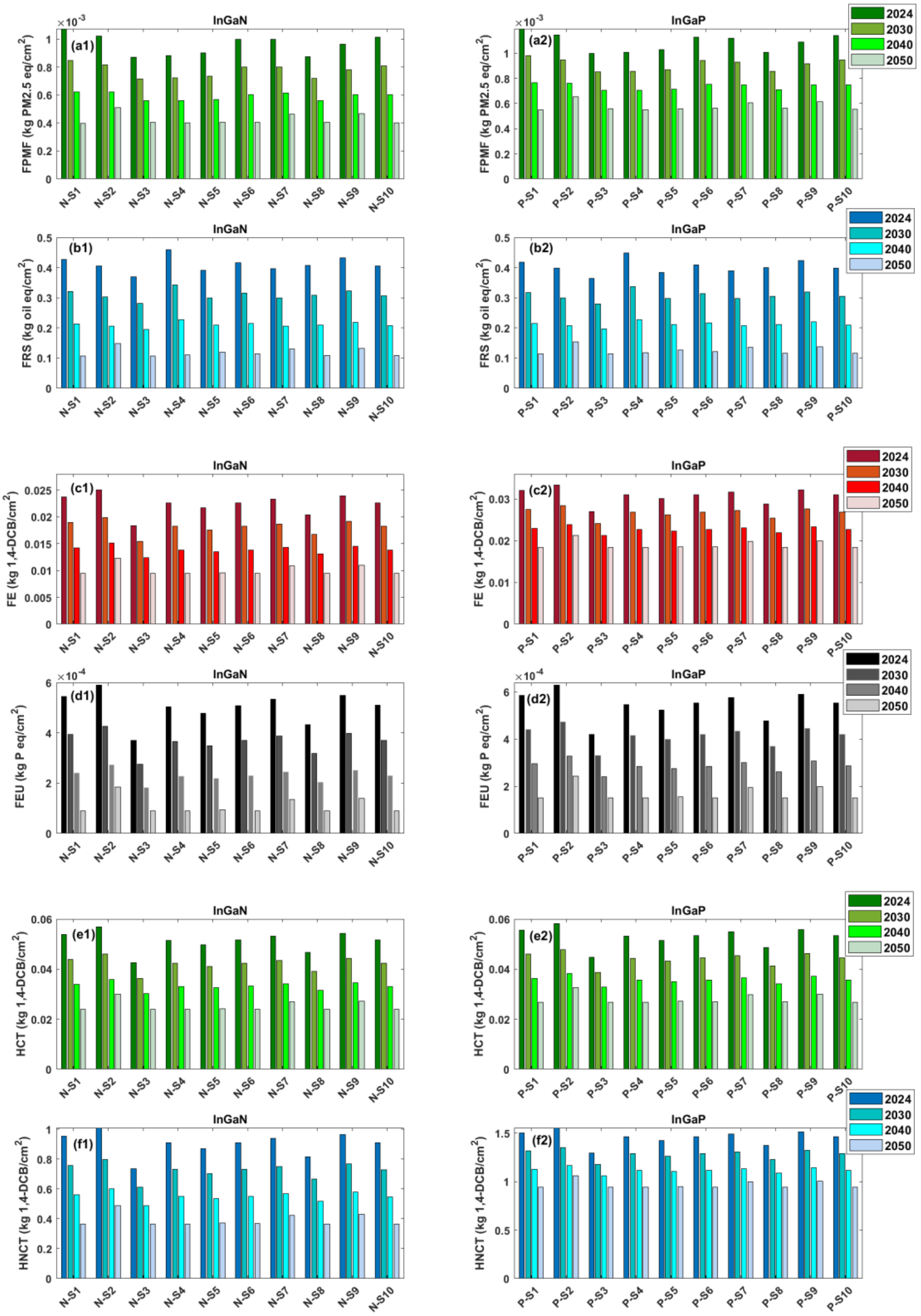

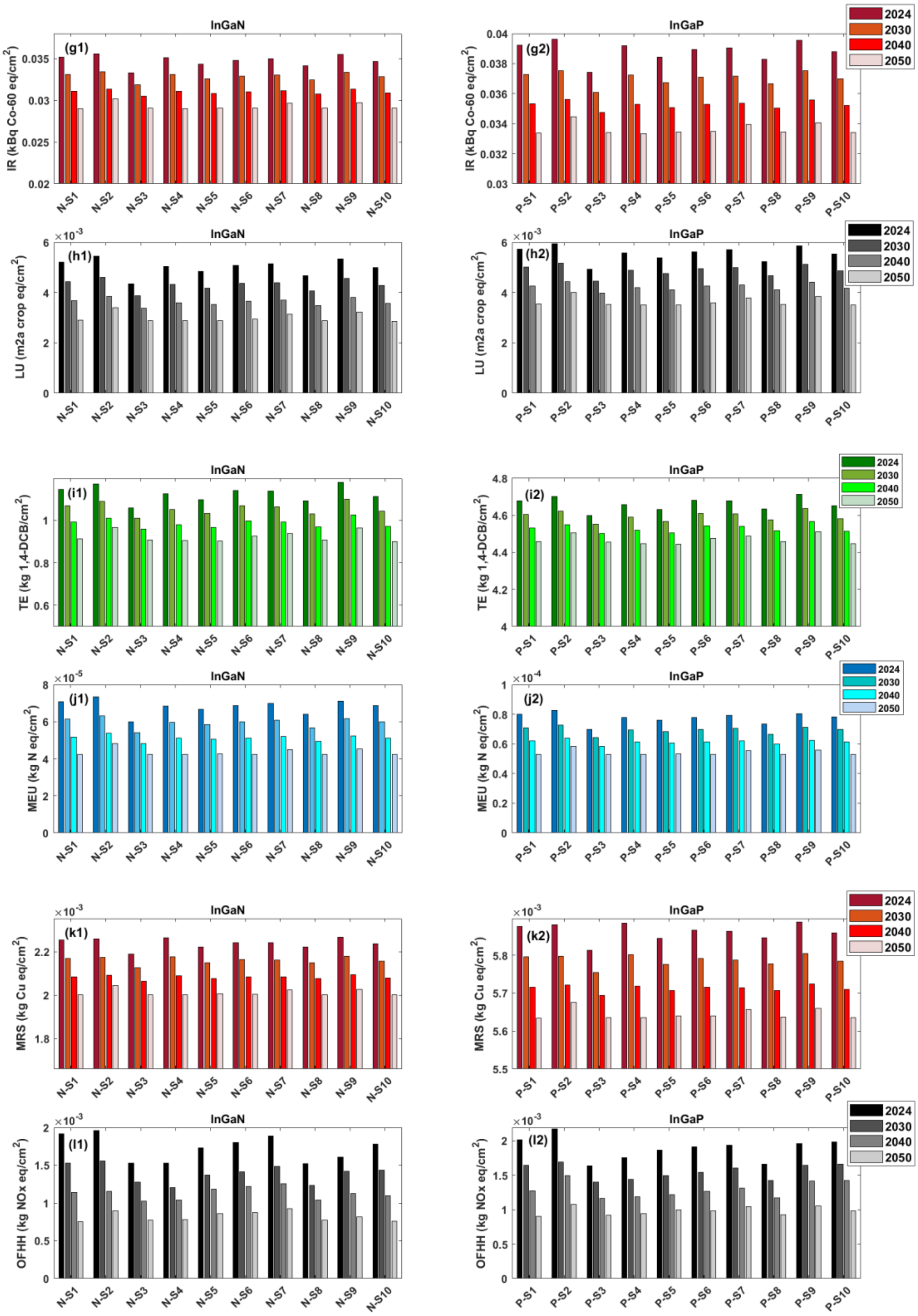
InGaN
InGaP
(g1)
(g2)
IR (kBq Co-60 eq/cm^2)
(h1)
(h2)
LU (m2a crop eq/cm^2)
(i1)
(i2)
TE (kg 1,4-DCB/cm^2)
(j1)
(j2)
MEU (kg N eq/cm^2)
(k1)
(k2)
MRS (kg Cu eq/cm^2)
(l1)
(l2)
OFHH (kg NOx eq/cm^2)
2024
2030
2040
2050
N-S1 N-S2 N-S3 N-S4 N-S5 N-S6 N-S7 N-S8 N-S9 N-S10
P-S1 P-S2 P-S3 P-S4 P-S5 P-S6 P-S7 P-S8 P-S9 P-S10

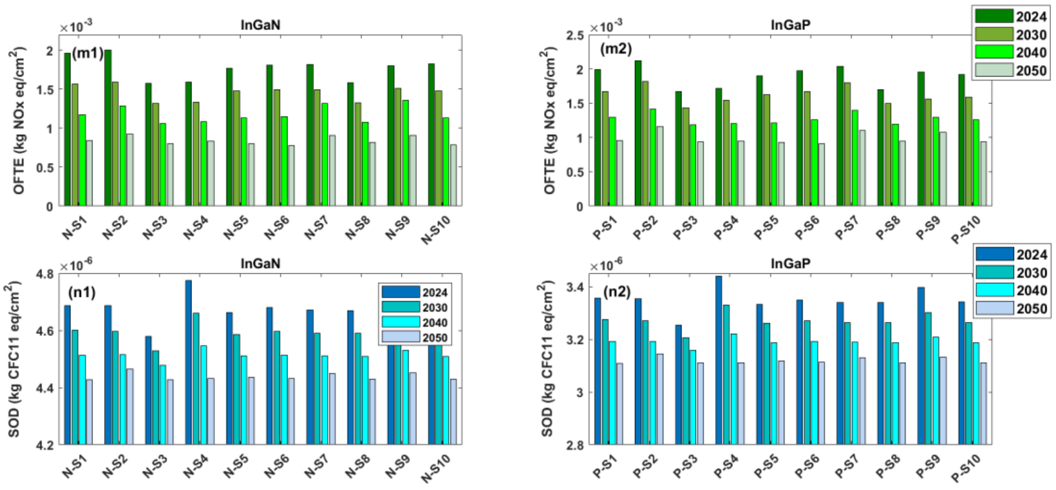

Fig S1. Projected environmental impact for InGaN and InGaP semiconductors across 14 categories from 2024 to 2050 comparing FPMF, FRS, FE, FEU, HCT, HNCT, IR, LU, ME, MEU, MRS, OFHH, OFTE, SOD results from the 80 international supply chain scenarios. (a1) shows FPMF impact results for InGaN, (a2) shows FPMF impact results for InGaP, (b1) shows FRS results for InGaN, (b2) shows FRS results for InGaP, (c1) shows FE results for InGaN, (c2) FE results for InGaP, (d1) shows FEU results for InGaN, (d2) shows FEU results for InGaP, (e1) represents HCT results for InGaN, (e2) represents HCT results for InGaP, (f1) shows HNCT results for InGaN, (f2) shows HNCT results for InGaP, (g1) shows IR results for InGaN, (g2) shows IR results for InGaP, (h1) shows LU results for InGaN, (h2) shows LU results for InGaP, (i1) shows TE results for InGaN, (i2) shows TE results for InGaP, (j1) shows MEU results for InGaN, (j2) shows MEU results for InGaP, (k1) shows MRS results for InGaN, (k2) shows MRS results for InGaP, (l1) shows OFHH results for InGaN, (l2) shows OFHH results for InGaP, (m1) shows OFTE results for InGaN, (m2) shows OFTE results for InGaP, (n1) shows SOD results for InGaN, and (n2) shows SOD results for InGaP.

Environmental impact breakdown by process

Fig S2 shows the impact contributions from the 8 processes (clean room, epitaxial growth, substrate preparation, etching, metal deposition, photolithography, testing and packaging, transportation) for four impact categories (WP, ME, TA, WD) for both technologies (InGaN and InGaP). As the differences in portions across scenarios are minimal, the interpretation is presented for only one scenario here (N-S6 and P-S6). However, the portion variations differ across impact categories. Figs S2a to S2f show the portion results for the GW impact category. The GW impact results show that for both InGaN and InGaP SCs, the cleanroom process currently dominates emissions but declines significantly by 2050 due to decarbonisation trends, particularly in fabrication-intensive countries like Taiwan and South Korea. In contrast, epitaxial growth and substrate production increase over time, becoming major contributors by 2050, especially for InGaP where epitaxy alone accounts for nearly 39% of total impact. Notably, transportation and testing and packaging (in Malaysia) remain low contributors throughout, reflecting their relatively minor environmental burden in the overall lifecycle.

Figs S2g to S2l illustrate the portion results for the ME impact category. For InGaN, the cleanroom process shows a steep decline by 2050 due to anticipated energy decarbonisation in fabrication regions, while testing and packaging (in Malaysia) and photolithography become increasingly significant contributors. In contrast, InGaP is heavily dominated by epitaxial growth, which rises steadily and accounts for over 64% of ME impact by 2050, overshadowing all other processes. Despite regional differences, transportation remains negligible in both cases throughout the timeline.

Figs S2m to S2r display the portion results for the TA impact category. For InGaN, the substrate process becomes the dominant contributor by 2050, growing steadily to account for nearly 40% of total impact, while the cleanroom impact drops sharply due to decarbonisation. In InGaP, epitaxial growth emerges as the primary hotspot, reaching over 37% by 2050, followed by a growing contribution from substrate production. This trend is primarily due to the energy-intensive nature of these processes, which relies heavily on high-temperature and high-purity processing Transportation impact remains low but increases steadily over time in both cases, particularly for InGaP.

Figs S2s to S2x show the portion results for the WD impact category. For both compound semiconductors, the cleanroom process starts as the major contributor but declines significantly by 2050 due to improvements in water efficiency and decarbonisation in fabrication hubs. Over time, epitaxial growth and substrate production become the leading contributors, reflecting their inherently high water demand for crystal growth and wafer processing. Metal deposition also shows a steady increase, while testing, packaging, and transportation remain minor throughout the lifecycle.

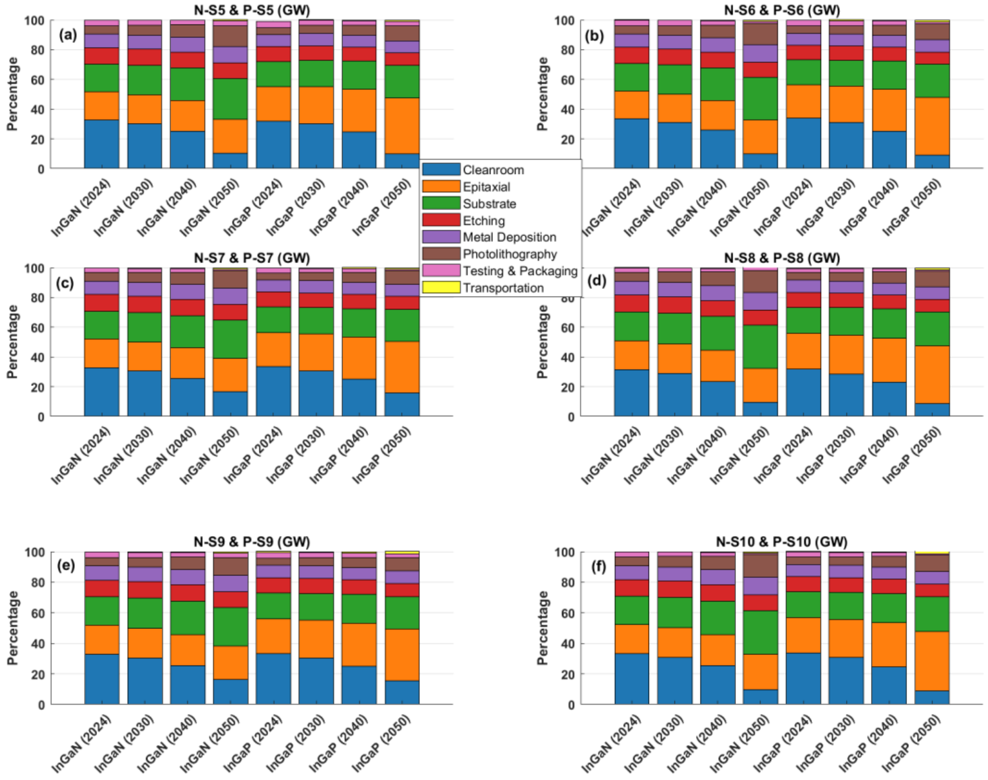

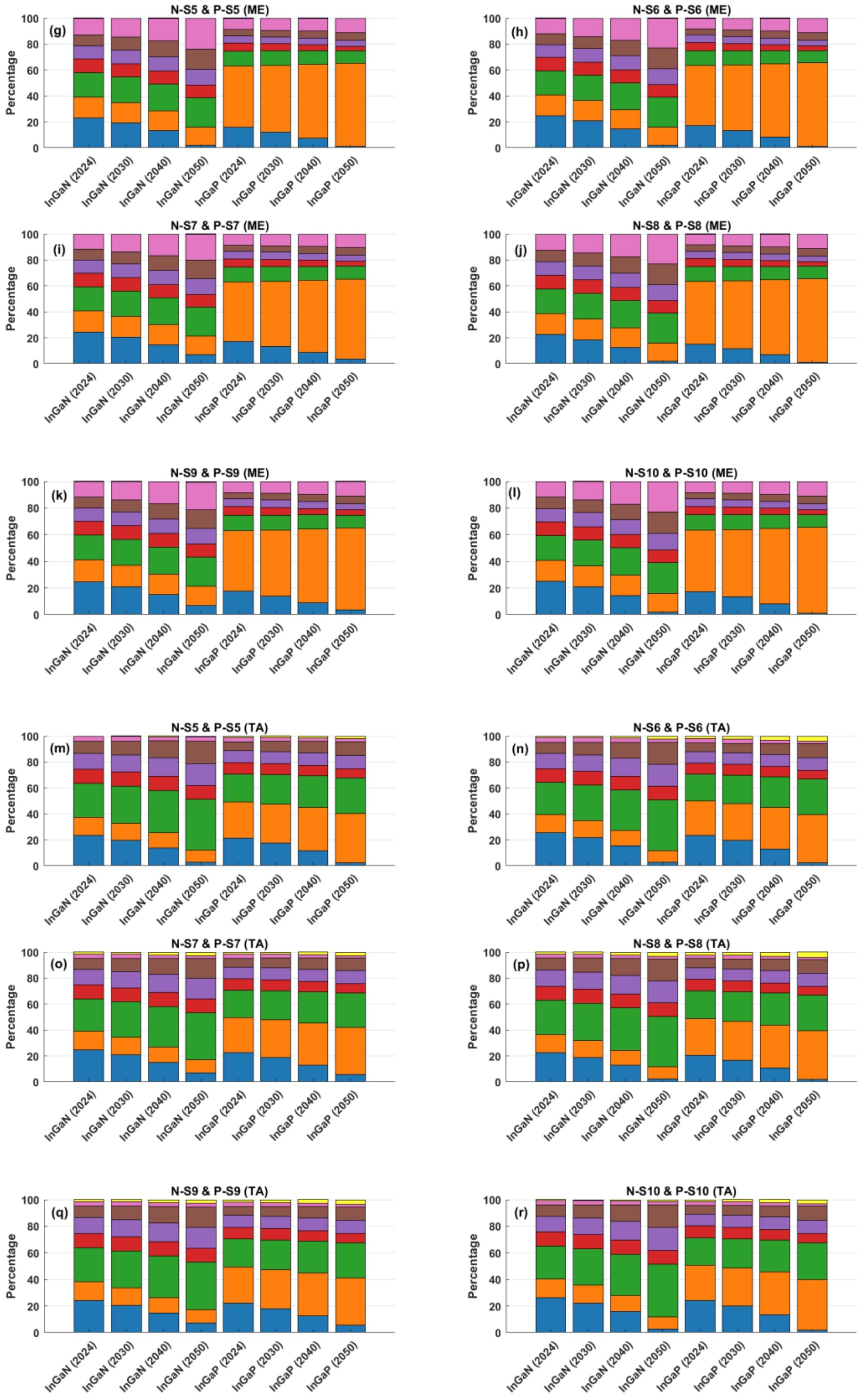
(g)
N-S5 & P-S5 (ME)
(h)
N-S6 & P-S6 (ME)
(i)
N-S7 & P-S7 (ME)
(j)
N-S8 & P-S8 (ME)
(k)
N-S9 & P-S9 (ME)
(l)
N-S10 & P-S10 (ME)
(m)
N-S5 & P-S5 (TA)
(n)
N-S6 & P-S6 (TA)
(o)
N-S7 & P-S7 (TA)
(p)
N-S8 & P-S8 (TA)
(q)
N-S9 & P-S9 (TA)
(r)
N-S10 & P-S10 (TA)
Percentage
0
20
40
60
80
100
InGaN (2024)
InGaN (2030)
InGaN (2040)
InGaN (2050)
InGaP (2024)
InGaP (2030)
InGaP (2040)
InGaP (2050)

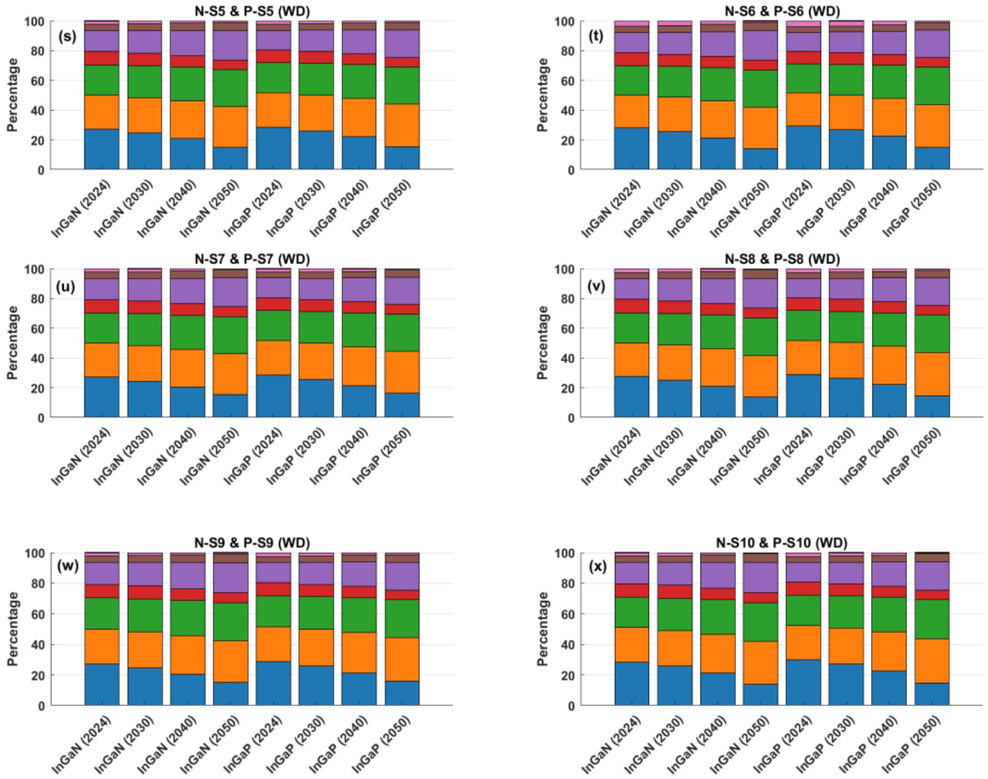

Fig S2. Impact contributions from 8 processes for InGaN and InGaP compound semiconductors for scenarios N-S5 to N-S10 and P-S5 to P-S10 comparing the WP, ME, TA and WD results between InGAN and InGaP from 2024 to 2050, Plots S2a to S2f represent the GW portion related to each supply chain, Plots S2g to S2l represent the ME portion related to each supply chain process, Plots S2m to S2r represent the TA portion related to each supply chain process, Plots S2s to S2x represent the WD portion related to each supply chain process

Sustainability performance rankings

Fig S3 illustrates the sustainability ranking results for 14 impact categories based on the geo-spatial prospective LCA results for InGaN and InGaP compound semiconductors for all international supply chain scenarios in 2024 and 2050.

Fine Particulate Matter Formation (FPMF)

In 2024, the best-performing scenarios in terms of FPMF remain primarily within the InGaN (N-S#) group. N-S3 (20) and N-S8 (19) lead with the highest scores, followed by N-S4 (18), N-S5 (17), and N-S9 (16) - indicating lower emissions of harmful fine particulates. The InGaP (P-S#) scenarios show weaker performance, especially P-S1 (1), P-S2 (2), and P-S10 (3), suggesting high particulate-related impact in those supply chains. By 2050, most InGaN scenarios show strong improvement - N-S1 rises to 20, and N-S3, N-S4, N-S5, N-S6, N-S8, and N-S10 maintain high scores (≥18), reflecting sustained progress in emission control or cleaner energy inputs. However, some InGaP routes remain problematic: P-S2 drops to 1, and P-S9 (3) and P-S7 (4) still lag, indicating persistent particulate pollution concerns in certain supply chain configurations.

Fossil Resource Scarcity (FRS)

In 2024, FRS scores reflect substantial variability across both InGaN and InGaP options. InGaP scenario P-S3 (score: 20) and InGaN scenario N-S3 (19) top the list, indicating minimal dependence on fossil energy or rare resource inputs. However, certain scenarios, notably N-S4 (score: 1) and N-S9 (3), reveal critical sustainability concerns. Encouragingly, the 2050 outlook shows strong gains among many InGaN scenarios, particularly N-S3 (maintaining 20) and N-S1 (jumping from 4 to 19), suggesting the adoption of cleaner energy or materials over time. That said, not all scenarios show progress - P-S2 declines to a score of 1 by 2050, marking it as a persistent supply chain sustainability hotspot.

Freshwater Ecotoxicity (FE)

FE scores in 2024 highlight the environmental burden of toxic substance release in various supply chain configurations. N-S3 again stands out with a perfect score of 20, joined by N-S8 (19), N-S5 (18), and N-S6 (17), all suggesting a relatively benign ecological profile. Conversely, P-S2 (1) and P-S1 (3) exhibit the highest ecological toxicity impacts, underlining potential risks to freshwater ecosystems. By 2050, most InGaN scenarios converge near the top end of the scale, with scores ranging between 17 and 20, reflecting effective mitigation of ecotoxic discharges. In contrast, most InGaP scenarios remain low-scoring (1–4), showing little to no improvement, and suggesting that toxic substance management remains a significant challenge in these pathways.

Freshwater Eutrophication (FEU)

FEU, driven by nutrient discharges, shows a similar pattern. In 2024, N-S3 once again takes the lead (score: 20), with strong support from N-S8 (18), N-S5 (16), and P-S3 (19). At the opposite end, P-S2 (1), N-S2 (2), and P-S9 (3) score poorly, indicating potential contributions to eutrophication in lakes and rivers. Looking ahead to 2050, InGaN scenarios show impressive gains, with N-S1, N-S3, and N-S4 all reaching scores of 19 or 20. InGaP scenarios remain far more static; P-S2, for example, remains at the lowest score of 1. A few, such as P-S5 and P-S8, show modest improvements, but the majority stay below score 10, pointing to persistent nutrient discharge issues in their life cycles.

Human Carcinogenic Toxicity (HCT)

In 2024, the scenario with the highest performance in terms of minimising HCT is N-S3 (score: 20), followed by P-S3 (19) and N-S8 (18). These scenarios are likely characterised by cleaner processing routes or the use of less hazardous materials. On the other hand, P-S2 (score: 1) and P-S9 (3) perform very poorly, pointing to elevated risks of carcinogenic exposure throughout their supply chains. By 2050, there is substantial progress within InGaN scenarios, particularly N-S1 (from 7 to 20), N-S4 (14 to 19), and N-S10 (12 to 19). Several InGaP scenarios, however, show stagnant or minimal improvement - for instance, P-S2 remains at the bottom (1), while many others cluster at a mediocre score of 10, implying limited advancements in reducing carcinogenic impacts.

Human Non-Carcinogenic Toxicity (HNCT)

Performance for HNCT in 2024 is also led by N-S3 (20), N-S8 (19), and N-S5 (18) - consistent with their strong performance across other health impact indicators. Meanwhile, P-S2 (1) and P-S9 (2) rank the lowest, suggesting concerns over exposure to toxic substances such as heavy metals or solvents. Encouragingly, by 2050, most InGaN routes maintain or improve their

performance - for instance, N-S10 improves from 16 to 20, while N-S3 and N-S4 remain at or near the top. In contrast, InGaP scenarios show very little change; P-S2 still scores just 1, and many others remain locked at 3 or below, pointing to persistent gaps in chemical safety or emissions control in these systems.

Ionizing Radiation (IR)

The 2024 data show N-S3 (20) and N-S8 (19) as the top performers with respect to minimising IR impacts, suggesting energy sourcing or facility-specific differences that lower radioactive emissions. P-S2 (1) and P-S9 (2) again rank worst, which might reflect supply chain reliance on nuclear-derived electricity or radiative materials. By 2050, InGaN scenarios such as N-S4, N-S5, and N-S6 all score 19 or 20, indicating widespread adoption of low-radiation technologies or cleaner grid electricity. Unfortunately, InGaP pathways continue to struggle: P-S2 remains at the lowest score (1), while others mostly score 4 or below, showing persistent exposure to ionizing sources.

Land Use (LU)

LU performance in 2024 is topped by N-S3 (20) and N-S8 (19), reflecting minimal land transformation, possibly due to higher efficiency or lower infrastructure demand. InGaP routes again lag behind: P-S2 (1) and P-S9 (2) show high environmental land footprints, potentially due to mining, processing, or energy generation-related land conversion. By 2050, there is a notable improvement among InGaN scenarios, with N-S10 reaching 20, and N-S3, N-S4, N-S5, and N-S8 all scoring 19, suggesting a maturing supply infrastructure with a smaller land footprint. InGaP routes do improve somewhat (e.g., P-S1 rises from 3 to 8), but most remain in the mid or lower range. P-S2 and P-S9, in particular, continue to underperform.

Marine Eutrophication

In 2024, MEU performance is highest for N-S3 (score: 20) and N-S8 (19), followed closely by N-S5 (18) and N-S4 (17). These scenarios demonstrate lower nitrogen emissions and runoff into aquatic systems, likely due to cleaner production processes or more efficient waste management. In contrast, P-S2 (1) and P-S9 (2) again rank lowest, suggesting nutrient-rich effluent discharge or wastewater mismanagement. By 2050, several InGaN scenarios such as N-S10 (20) and N-S3, N-S4, and N-S8 (each scoring 19) maintain or enhance their performance, indicating consistent mitigation of eutrophication potential. However, most InGaP scenarios stagnate, clustered around a low-to-moderate score of 5 or below, suggesting minimal progress in reducing marine nutrient pollution.

Mineral Resource Scarcity

Regarding mineral resource use in 2024, N-S3 (score: 20) and N-S5, N-S8 (19) are the top performers, reflecting efficient material use, circularity, or sourcing of abundant alternatives. All InGaN scenarios (N-S#) perform well in this category, scoring 17 or above, suggesting a generally strong mineral efficiency across these supply configurations. In stark contrast, InGaP routes (P-S#) are consistently poor - P-S2 and P-S10 score just 1 and 3, respectively. This suggests heavier reliance on scarce or critical raw materials such as gallium or indium without mitigation strategies. By 2050, InGaN performance becomes nearly uniform and excellent, with most scenarios achieving scores of 19–20. InGaP scenarios remain underwhelming: P-S2

remains at 1, and most others stagnate around score 4 or 5, indicating a continued vulnerability to critical material dependence and supply risk.

Ozone Formation – Human Health

In 2024, N-S3 (score: 20) and N-S8 (19) again lead in minimising precursors to ground-level ozone, reducing respiratory health risks. These are followed closely by N-S4 (18) and N-S9 (17), indicating lower emissions of volatile organic compounds (VOCs) or NOx. Conversely, P-S2 (1) and P-S10 (3) reflect elevated emissions that contribute to human ozone exposure. By 2050, N-S3 and N-S8 maintain high scores (20 and 19, respectively), and N-S10 improves from 12 to 16, suggesting continuous emission reduction. Several InGaP scenarios, however, show modest improvement, P-S1 improves from 2 to 8, but P-S2 remains at 1, and others still score below 5, indicating an urgent need to address air quality-related emissions in InGaP production.

Ozone Formation – Terrestrial Ecosystems

For 2024, the terrestrial ecosystem impacts of ozone are lowest (i.e., most sustainable) in N-S3 (20) and N-S8 (19), indicating strong performance in reducing emissions that contribute to vegetation damage. These are followed by N-S4 (18) and N-S5 (14). P-S2 (1) again scores worst, with others like P-S1 and P-S7 also performing poorly. By 2050, N-S3 and N-S8 remain at the top (20 and 19, respectively), and N-S10 improves significantly from 10 to 17, showing ecosystem-sensitive design. While InGaP scenarios show small gains - e.g., P-S1 improves from 4 to 6, and others like P-S3, P-S4, and P-S8 stabilize at 7. Their overall impact on terrestrial ozone formation remains a concern, particularly given continued low scores for P-S2 and P-S7.

Stratospheric Ozone Depletion

In 2024, InGaP supply chains (P-S#) demonstrate substantially better performance in avoiding substances that deplete the ozone layer. P-S3 scores the maximum of 20, with P-S2, P-S5, P-S6, P-S7, P-S8, and P-S10 all scoring 19, and others like P-S1, P-S4, and P-S9 following closely behind. These high scores suggest that the InGaP pathways either avoid ozone-depleting substances or incorporate cleaner processes that limit emissions of substances like halocarbons. In stark contrast, all InGaN scenarios score between 1 and 4, with N-S4 scoring the lowest (1) and others like N-S3 and N-S10 managing just 4 and 3, respectively. This poor performance may be attributed to refrigerant use, chemical feedstocks, or process-related emissions not yet mitigated in the InGaN supply chain. By 2050, there is a slight improvement among InGaN pathways (for instance, N-S1, N-S3, N-S4, N-S6, and N-S8 reach scores of 4 or 5) but the progress remains limited. Notably, N-S2 drops to 1, and most others linger in the very low 2-4 range. In contrast, InGaP routes remain strong: P-S1 improves to a perfect 20, and P-S4 and P-S10 consistently maintain scores of 19, suggesting a sustained commitment to ozone-depleting substances-free production and maintenance processes. These results indicate that, at least in this impact category, InGaP production is far more ozone-safe than InGaN, and the gap remains wide even in future projections.

Terrestrial Ecotoxicity

In 2024, the InGaN scenarios excel in limiting toxic emissions that impact TE. N-S3 leads with a score of 20, closely followed by N-S5 (19), and N-S1, N-S4, N-S6, N-S7, and N-S9 all score 18, with even the lowest (N-S2, N-S8) at 17 - showing remarkable consistency. This suggests effective control over heavy metals, persistent organic pollutants, or hazardous by-products, likely

through material selection, cleaner process chemicals, and controlled emissions. Conversely, InGaP scenarios perform poorly in 2024. P-S9 (score: 1) and P-S2 (2) reflect potentially severe ecosystem toxicity risks, while even the best among them, P-S3 (6), remains well below any of the InGaN scenarios. This may result from the use of more toxic precursors or inefficient mitigation of process waste. In 2050, the strong performance of InGaN scenarios continues, with N-S10 improving to 20 and N-S4 and N-S8 rising to 19. Most other scenarios maintain their high scores (17–19), reflecting long-term improvements in managing ecotoxic emissions. InGaP scenarios, on the other hand, see only marginal improvements: P-S1 increases from 3 to 4, and P-S5 and P-S4 improve slightly, but the worst cases (like P-S9 (still 1) and P-S2 (still 2)) remain deeply problematic. This highlights a persistent ecological burden associated with InGaP supply chains, likely due to material and waste-related toxicity not fully addressed over time.

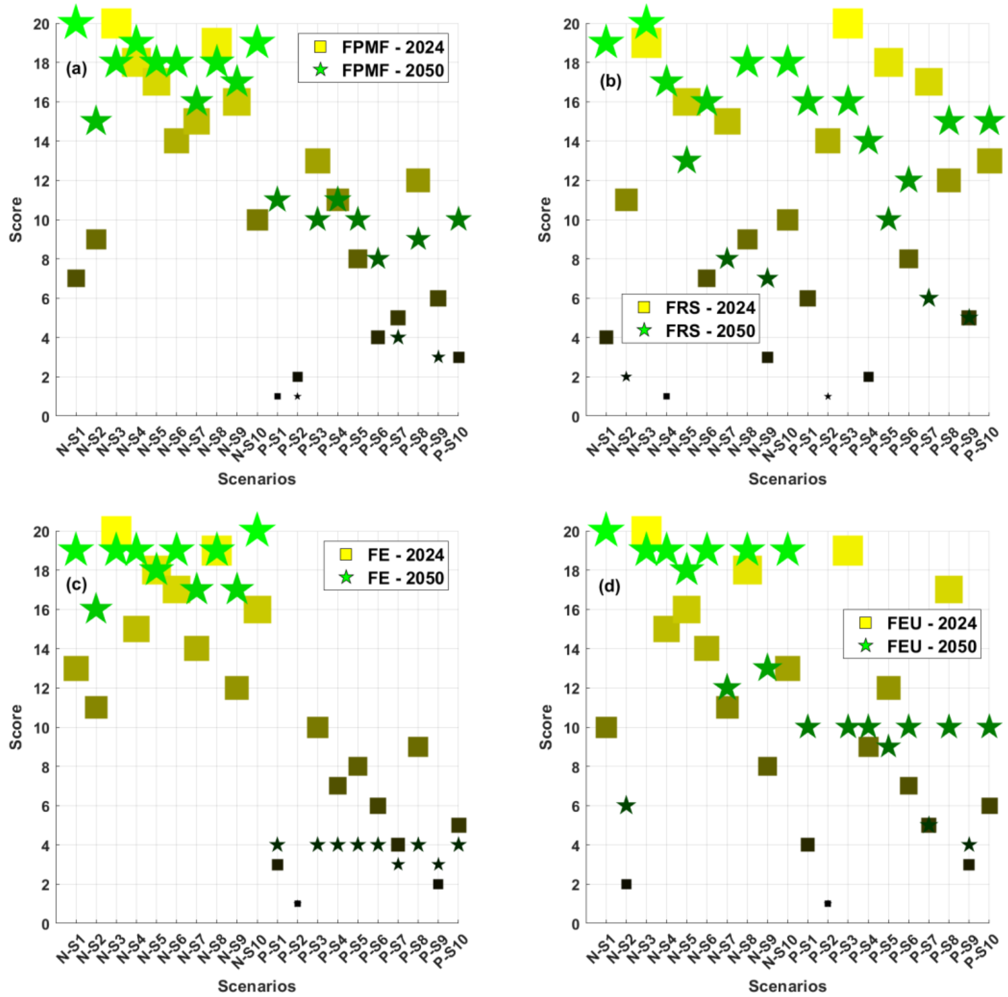

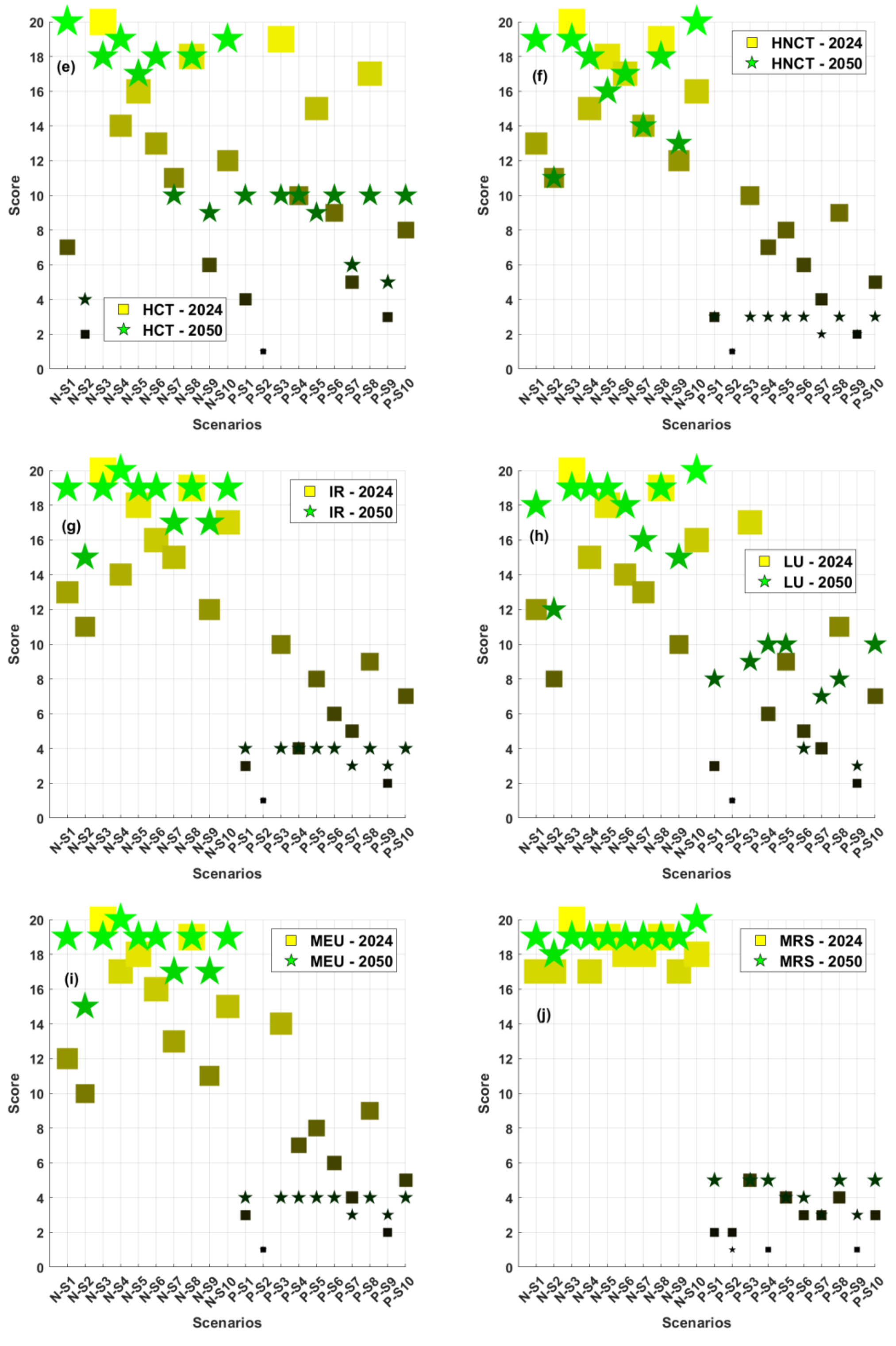
(e)
HCT - 2024
HCT - 2050
(f)
HNCT - 2024
HNCT - 2050
(g)
IR - 2024
IR - 2050
(h)
LU - 2024
LU - 2050
(i)
MEU - 2024
MEU - 2050
(j)
MRS - 2024
MRS - 2050
Score
Scenarios
N-S1 N-S2 N-S3 N-S4 N-S5 N-S6 N-S7 N-S8 N-S9 N-S10 P-S1 P-S2 P-S3 P-S4 P-S5 P-S6 P-S7 P-S8 P-S9 P-S10
0 2 4 6 8 10 12 14 16 18 20

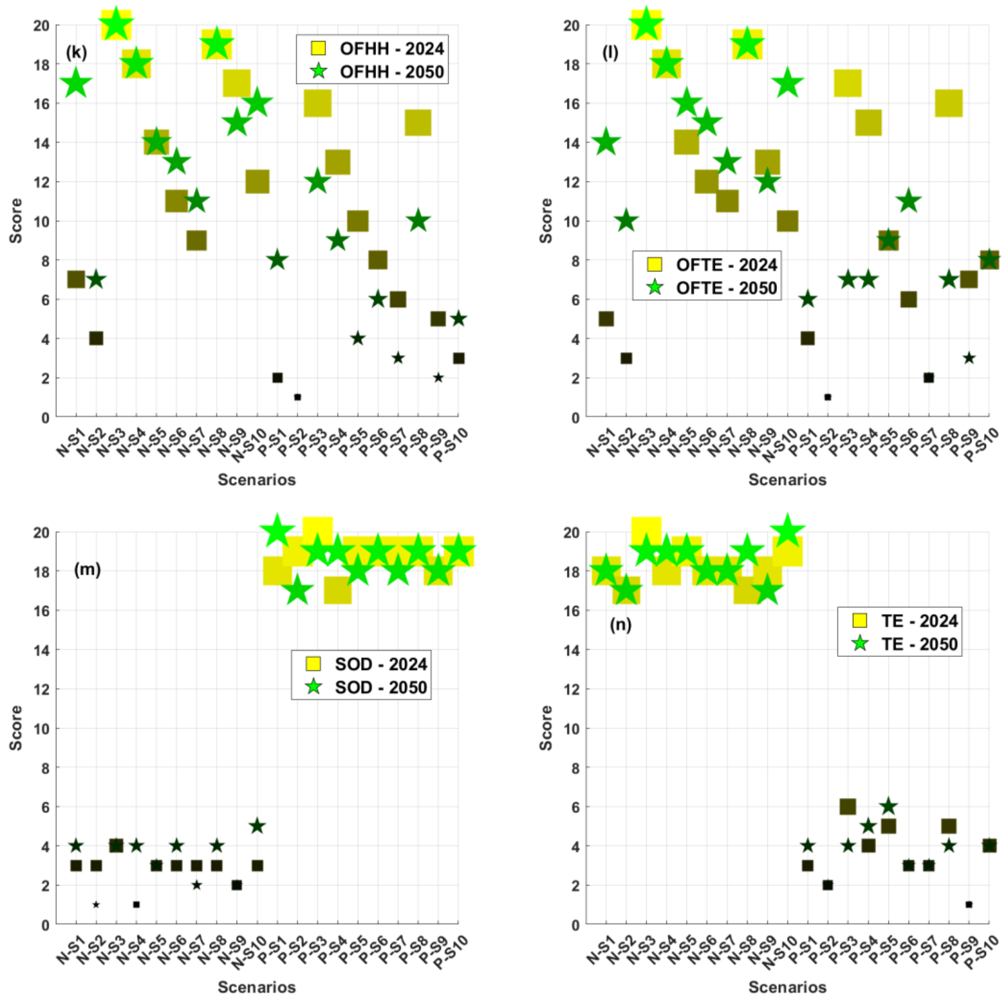

Fig S3. Sustainability ranking results by 14 indicators (FPMF, FRS, FE, FEU, HCT, HNCT, IR, LU, MEU, MRS, OFHH, OFTE, SOD, TE) for InGaN and InGaP across all international supply chain scenarios in 2024 and 2050. (a) Ranking results for FPMF, (b) Ranking results for FRS, (c) Ranking results for FE, (d) Ranking results for FEU, (e) Ranking results for HCT, (f) Ranking results for HNCT, (g) Ranking results for IR, (h) Ranking results for LU, (i) Ranking results for MEU, (j) Ranking results for MRS, (k) Ranking results for OFHH, (l) Ranking results for OFTE, (m) Ranking results for SOD, and (n) Ranking results for TE

Table S1 presents the results of a sensitivity analysis on the GW impact category, showing the effect of ±10% variations in key input parameters. The results indicate that energy consumption is the most influential factor, with a ±10% change leading to a 6.8% variation in GW impacts. Variations in chemicals and solvents result in a smaller 2.1% change, while gaseous precursors contribute minimally (0.3%). Metals show a modest impact of 1.5%. Overall, these findings confirm that typical ±10% fluctuations in material and energy inputs produce only minor changes in the total GW impact, supporting the robustness of the LCA results presented in this study.

Table S1. Sensitivity of Global Warming impacts to ±10% variations in key input parameters

Parameter	Change in value	GW Variation (%)
Gaseous	±10 %	±0.3 %
Chemicals and solvents	±10 %	±2.1 %
Metals	±10 %	±1.5 %
Energy	±10 %	±6.8 %

Approximate aggregated percentage distributions of chemicals/gases, energy, and water across the main processing stages are presented in Table S2 to enhance transparency while maintaining confidentiality constraints.

Table S2. Approximate aggregated inventory distribution by major process

Process	Chemicals & Gases (%)	Energy (%)	Water (%)
Substrate preparation	30	25	30
Epitaxial growth	35	35	35
Fabrication	35	40	35
